# Adhesion, Biofilm Formation, and *luxS* Sequencing of *Campylobacter jejuni* Isolated From Water in the Czech Republic

**DOI:** 10.3389/fcimb.2020.596613

**Published:** 2020-11-16

**Authors:** Ekaterina Shagieva, Martin Teren, Hana Michova, Nicol Strakova, Renata Karpiskova, Katerina Demnerova

**Affiliations:** ^1^ Laboratory of Food Microbiology, Department of Biochemistry and Microbiology, University of Chemistry and Technology, Prague, Czechia; ^2^ Laboratory of Zoonoses and Antibiotic Resistance, Department of Microbiology and Antimicrobial Resistance, Veterinary Research Institute, Brno, Czechia

**Keywords:** *Campylobacter jejuni*, biofilm, adhesion, *luxS*, foodborne pathogen, confocal laser scanning microscopy, water

## Abstract

The microaerophilic pathogen *Campylobacter jejuni* is a leading bacterial cause of human gastroenteritis in developed countries. Even though it has a reputation as a fastidious organism, *C. jejuni* is widespread and can be easily isolated from various animals, food, and environmental sources. It is suggested that an ability to form biofilms is probably necessary for the survival of *C. jejuni* under harsh environmental conditions. The first step required for successful biofilm formation is adhesion to a suitable surface. Therefore, in this work, the degree of adhesion was evaluated, followed by characterization and quantification of biofilms using confocal laser scanning microscopy (CLSM). A total of 15 isolates of *C. jejuni* were used in the experiments (12 isolates from surface and waste waters, 1 human clinical, 1 food and 1 ACTT BAA-2151 collection strain, all samples originated from the Czech Republic). Regardless of the sample origin, all *C. jejuni* isolates were able to adhere to the polystyrene surface within 30 min, with the number of attached cells increasing with the time of incubation. The resulting data showed that all isolates were able to form complex voluminous biofilms after 24 h of cultivation. The average amount of biovolume ranged from 3.59 × 10^6^ µm^3^ to 17.50 × 10^6^ µm^3^ in isolates obtained from different sources of water, 16.79 × 10^6^ µm^3^ in the food isolate and 10.92 × 10^6^ µm^3^ in the collection strain. However, the highest amount of biomass was produced by the human clinical isolate (25.48 × 10^6^ µm^3^). Similar to the quantity, the architecture of the biofilms also differed, from a rugged flat monolayer of cells to large clustered structures. Further, all isolates were tested for the presence of the *luxS* gene, as the *luxS*/AI-2 (autoinducer-2) quorum sensing pathway has been previously connected with enhanced biofilm formation. Two isolates originated from surface waters did not possess the *luxS* gene. These isolates formed thinner and sparser biofilms lacking the presence of significant clusters. However, the ability to adhere to the surface was preserved. The sequencing of the *luxS*-containing fragments shown a high similarity of the *luxS* gene among the isolates.

## Introduction

Campylobacteriosis is an infection caused by *Campylobacter* spp., which is considered one of the main causes of foodborne gastrointestinal bacterial infections worldwide (Allos, 2001). According to the latest EFSA report, in total 246,571 cases of campylobacteriosis were confirmed in 36 EU countries in 2018. Interestingly, the Czech Republic is among the countries with the highest incidence of the disease (215.8 cases per 100,000 inhabitants) (EFSA and ECDC, 2019). The most common species causing the human infection is *Campylobacter jejuni* (Kaakoush et al., 2015). Symptoms associated with the infection usually last two to five days and include diarrhea, vomiting, and abdominal pain (Black et al., 1988). The disease is usually self-limiting, but sometimes can result in serious autoimmune diseases, such as Guillain-Barré and Miller-Fischer syndromes, and reactive arthritis (Salloway et al., 1996; Ang et al., 2001; Pope et al., 2007).

Although campylobacteriosis belongs to bacterial zoonoses and is related to consumption of raw or undercooked meat especially from poultry and drinking of unpasteurized milk (Blaser et al., 1983; Humphrey, 1986; Sahin et al., 2001; Zimmer et al., 2003), the disease can also be disseminated also through the environment, in particular through contaminated water (Carter et al., 1987; Obiri-Danso and Jones, 1999; Daczkowska-Kozon and Brzostek-Nowakowska, 2001; Hörman et al., 2004).

It is known, that *C. jejuni* can survive in untreated or inadequately treated aquatic environments, including wells and groundwater (Stanley et al., 1998). Typically, contamination occurs directly through feces of wild animals or livestock, through wastewater from farms, slaughterhouses, manure, and even as a result of heavy rain (Sacks et al., 1986; Eberhart-Phillips et al., 1997; Clark et al., 2003). Therefore, it has been suggested that its survival in the water systems of animal husbandry facilities and animal-processing units contributes to the infection of animals, and cross-contamination of animal carcasses (Humphrey and Beckett, 1987; Pearson et al., 1993). Thus, the survival of *C. jejuni* in the aquatic environment is important both directly and indirectly in the occurrence of human diseases. There were several reports of how the water from the environment may pose a source of outbreaks of campylobacteriosis and in almost all cases, well or drinking water was contaminated with surface or wastewater (Gubbels et al., 2012; Bartholomew et al., 2014; Pedati et al., 2019).

Compared to many other foodborne pathogens, *C. jejuni* is demanding on environmental conditions, it multiplies under a microaerobic atmosphere (5% oxygen, 10% carbon dioxide and 85% nitrogen) at a temperature ranging between 37°C and 42°C (Park, 2002). Theoretically, these properties make *C. jejuni* incapable of existing outside the host in a natural aerobic environment (Park, 2002; Nguyen et al., 2012), but paradoxically, it not only survives in foods that are subjected to difficult processing conditions (preservation, temperature changes, stress, different pH), but can also be transmitted through natural sources (Klancnik et al., 2009).

Judging by the published research, one of the main strategies that *C. jejuni* uses to survive in the environment is the ability to attach to surfaces and form biofilms (Chmielewski and Frank, 2003). Biofilms are commonly defined as adherent microbial cells embedded within a matrix of extracellular polymeric substances (Costerton et al., 1995; Donlan and Costerton, 2002). It is known, that *C. jejuni* can adhere to both various inert surfaces (e.g. stainless steel, fiberglass, coverslips, nitrocellulose membranes, various plastics) and biotic surfaces (animal and human intestinal cell lines) (Pogacar et al., 2009; Sulaeman et al., 2012; Pogačar et al., 2015). Cell adhesion precedes the formation of biofilms, which represent a protection mechanism against environmental stresses, antimicrobial agents, and the host’s immune response (Hall-Stoodley et al., 2004; Blanpain-Avet et al., 2011). *C. jejuni* can also form biofilms on various abiotic surfaces commonly used in irrigation systems, such as acrylonitrile butadiene styrene and polyvinyl chloride plastics (Reeser et al., 2007). It also has the ability to form biofilms in water supply systems in livestock complexes and animal processing plants, which can then represent a constant source of infection for both animals and humans (Buswell et al., 1998; Zimmer et al., 2003).

The molecular background of biofilm formation in *Campylobacter* is still not fully understood, although there is evidence that flagella, surface proteins, and quorum sensing represented by S-ribosylhomocysteine lyase (*luxS*) are required to maximize the biofilm formation (Elvers and Park, 2002; Asakura et al., 2007; Kalmokoff et al., 2006; Kim et al., 2015). Several studies have already demonstrated that this gene is involved in a variety of physiological pathways in *C. jejuni*, including motility, autoagglutination, flagellar expression, oxidative stress, and animal colonization. It was also shown that *luxS*-deficient mutants form significantly fewer biofilms (Elvers and Park, 2002; Reeser et al., 2007; Šimunović et al., 2020).

Previous studies of *Campylobacter* spp. biofilms focused mainly on cultivation under standard laboratory conditions or under artificial stress (Reuter et al., 2010; Oh et al., 2016; Melo et al., 2017). Biofilms were mostly characterized by semiquantitative analysis using crystal violet, which generally provides a comparative characteristic of different isolates, however, it does not provide information about the biofilm structure (Gunther and Chen, 2009; Teh et al., 2010; Zhong et al., 2020). Studies that described structural elements of biofilms using CLSM (Sanders et al., 2007; Ica et al., 2012; Bronnec et al., 2016), in turn, did not perform a comparative characterization of biofilms formed by isolates with different backgrounds. As far as we know, the experiments were mostly examining isolates originating from various animal, food and clinical samples, but excluded environmental isolates, such as those isolated from water. Therefore, this study is focused on the comparison of isolates obtained from different sources (surface and waste water, food, and clinical samples), with an emphasis on their ability to adhere to a surface and subsequently form a biofilm. All isolates were also tested for the presence and the respective sequence of the *luxS* gene, to confirm its crucial role in biofilm development.

## Materials and Methods

### Bacterial Isolates and Culture Conditions

All *C. jejuni* isolates obtained from the environment (7 from the surface water, 5 from sewage, 1 form meat sample and 2 clinical human isolates) were collected from surface and waste water within the whole Czech Republic in the period of 2018 to 2019 (**Table 1**) were stored at −80°C in 20% glycerol with 80% Brain Heart Infusion (BHI; Oxoid, UK). They were routinely grown on Karmali agar (Oxoid, UK) at 42°C under microaerobic conditions (5% O_2_, 10% CO_2_, 85% N_2_) for 24 h in a multi-gas incubator MCO-18M (Sanyo, Japan).

**Table 1:** C. *jejuni* isolates used in this study.

Name	Origin	Source	The presence of the *luxS* gene
Cj5648P	Water	Pond, 2019	Yes
Cj5643P		Pond, 2019	Yes
Cj5683P		Pond, 2019	No
Cj5715P		Pond, 2019	Yes
Cj5654P		Pond, 2019	Yes
Cj5653P		Pond, 2018	No
Cj5650P		Pond, 2019	Yes
Cj5640W		Outlet of a wastewater treatment plant, 2019	Yes
Cj5623W		Outlet of a wastewater treatment plant, 2019	Yes
Cj5689W		Outlet of a wastewater treatment plant, 2019	Yes
Cj5629W		Outlet of a wastewater treatment plant, 2019	Yes
Cj5716W		Outlet of a wastewater treatment plant, 2018	Yes
Cj1M	Food	Butcher shop, 2019	Yes
Cj5718C	Clinical	Hospital, 2019	Yes
Cj81176		ATCC Collection (BAA-2151), originally from outbreak	Yes

### Biofilm Formation Assay

All biofilms were produced in 96-well polystyrene microtiter plate with μClear^®^ bottom (thickness of 190 ± 5 µm; Greiner Bio-one, Germany) under static conditions according to Turonova et al. (2015), with certain modifications mentioned below. Briefly, *C. jejuni* was grown 48 h on the Karmali agar, the cells were then resuspended in Mueller-Hinton Broth (MHB) to reach OD_600 nm_ = 0.8 ± 0.1. Resulting bacterial suspension was inoculated into sterile 96-well plates (250 µl per well) in technical triplicates and incubated at 42°C at microaerobic atmosphere for 2.5 h to allow the cells to adhere to the bottom of the well. After that, each bacterial suspension was carefully replaced with 250 µl of fresh sterile MHB. After 24 h of incubation at 42°C, all wells were carefully washed 3 times with sterile physiological solution. At last, the wells containing 150 µl of physiological solution were stained by adding 50 µl of 5 µM Syto 9 (Invitrogen, USA) directly into the wells. The experiments were carried out in three independent biological replicates and contained a control of potential bacterial contamination (wells containing sterile medium).

### Confocal Laser Scanning Microscopy (CLSM)

The biofilm images were acquired with Olympus IX81F- ZDC2 (Olympus, Japan) confocal scanning laser microscope with spinning disc (CLSM), equipped with Ander IQ software (Andor, Belfast, UK) using an objective Clara 10x. All wells were first scanned manually in bright field to observe the biofilm structure, then one representative location was selected for the CLSM analysis. For evaluation of the 3D images of the biofilm structure and its volume, stacks of horizontal planar images with a z‑step selected according to the NY Quist sampling (3.57 μm) were recorded in the green channel (excitation 488 nm, emission 525 nm). The single snapshots of 1040 × 1392 pixels representing an area of 670.8 × 897.8 µm were analyzed by the IMARIS × 64 7.6.4 software, resulting in 3D model of the biofilm structure (Biplane, Switzerland). The volume of the model was then used as a parameter for comparison of the amount of the biofilms. The bio-volume corresponds to the total volume of cells and eDNA in the acquired field.

### Adhesion Assay

To evaluate the rate of bacterial adhesion, 96- well clear-bottom plates were inoculated with 100 μl of *C. jejuni* suspension in MHB (OD_600 nm_ = 0.8 ± 0.1). After selected time of incubation (30, 60, 120, 180 min) at 42°C under microaerobic conditions, Syto 9 was added to each well, allowing the cells to stain for 15 min. After the staining, the supernatants containing the non-adhered cells were removed from each well, and the wells were carefully rinsed three times with sterile distilled water and quickly dried in the air. Further, 100 µl of 1.5% low melting agarose (Sigma-Aldrich, USA) was added, in order to fix the bacteria attached to the bottom of the well. After solidification, plates were viewed with the CLSM using a water immersion objective 40x. The adhesion rate of the isolates was evaluated by counting the cells in ten different fields of each well. The experiment was performed in 3 biological and 3 technical replicates.

### PCR Confirmation and Cloning of the *luxS* Gene

The genomic DNA of tested isolates was isolated according to the protocol described by He (2011). The presence of the *luxS* gene was confirmed by PCR with primers specific to the inner region of the mentioned gene (primer set 1, **Table 2**). For the purpose of the sequence analysis, approximately 800 bp fragment containing the *luxS* gene was amplified with primer set 2 containing modified adaptors for restriction enzymes NcoI and EcoRI (**Table 2**). Subsequently, amplified fragment was cloned to the pGEM-T easy vector (Promega) *via* technique of the sticky ends. Subsequently, the ligation mixture was transformed into the competent cells of *Escherichia coli* DH5α (NEB, USA) by the routine heat-shock protocol described by Sambrook and Russell (2006). Positive colonies of each sample were selected on Lysogeny agar (LB-A) (Hi-media, India) containing ampicillin (100 µg/ml), X-Gal (40 µg/ml) and IPTG (50 µg/ml) (all from Merck, USA).

**Table 2:** Primers used in this study.

Primer		Sequence	Size (bp)	Restriction site
Set 1	seq_F	TTGATTTGCGTTTTTGCGTA	222 bp	NA
	seq_R	CTTTCATGGCTGCTTCCCAA	222 bp	NA
Set 2	pGEM_F	CG**CCATGG**GAGCATGAACTTCAAGACCT	800 bp	**NcoI**
	pGEM_R	AC**GAATTC**CAAAGGACGCACTAGATACT	800 bp	**EcoRI**

The restriction sites for NcoI and EcoRI highlighted in bold.

The plasmid containing the fragment of interest of each sample was isolated by the GenElute™ HP Plasmid Miniprep Kit (Merck, USA), sequenced and the data were deposited to the NCBI database.

### Statistical Analysis

The data were expressed as the mean ± standard deviation. Statistical analysis was carried out using Statistica 10.0. The significance level was chosen at 95%, consequently, an effect was considered significant if its p-value was lower than 0.05. The association between the ability to adhere and the ability to form biofilm was evaluated by Spearman correlation analysis.

Calculations and graphs were processed using Microsoft Excel 2016.

## Results

### Adhesion

**Figure 1:**
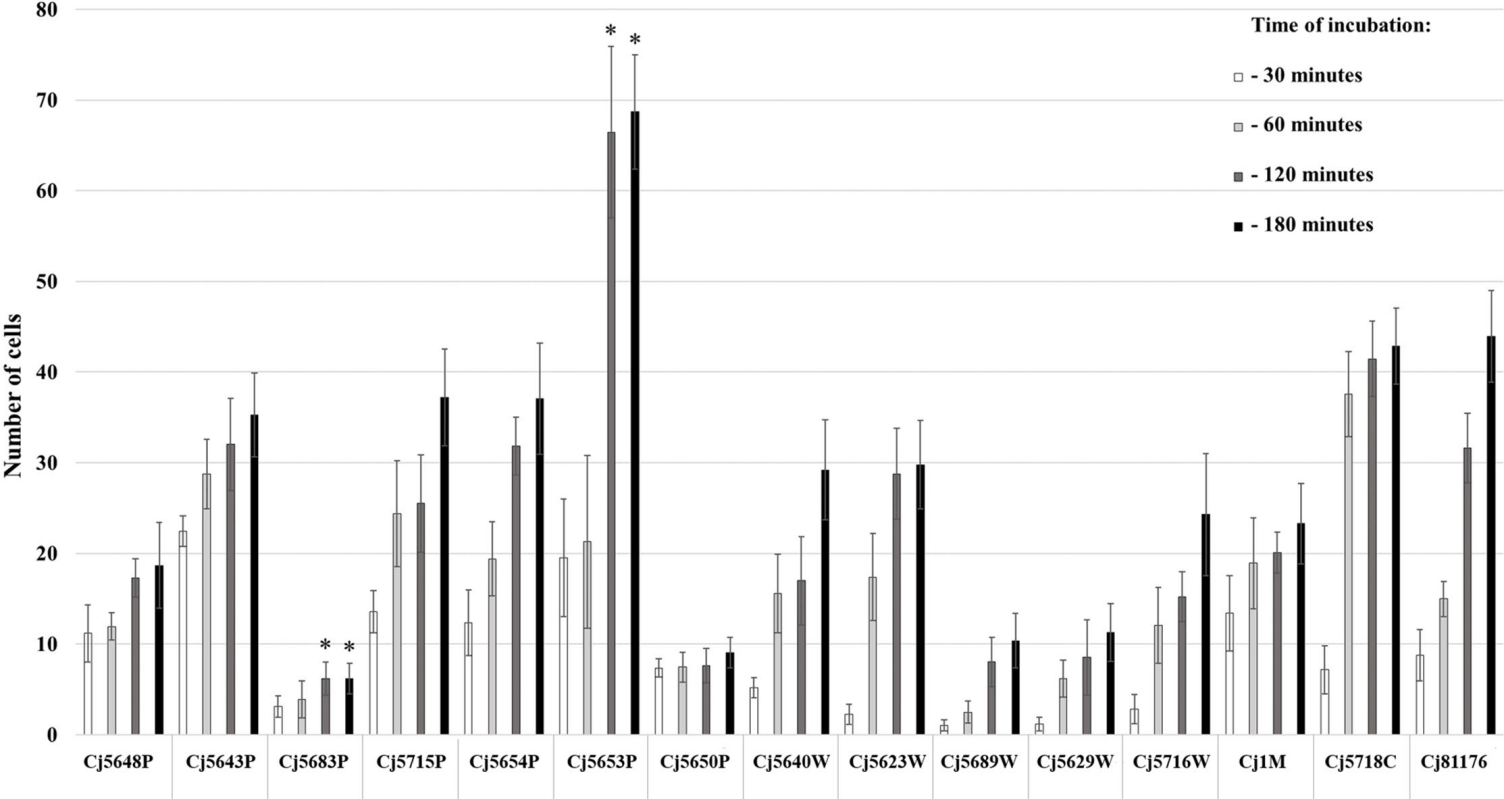
Adhesion capability of *Campylobacter jejuni* isolates measured at four different timepoints of incubation at 42°C under microaerobic atmosphere. The bars represent means of 9 values (triplicate of three independent cultures), the error bars represent standard deviation from the mean; * marks significantly different isolates (p < 0.05).

The ability of cells to adhere to the bottom of the microtiter plate was evaluated by counting the cells stained with Syto 9 after visualization on CLSM. The adhesion of 15 C*. jejuni* isolates was evaluated after 30, 60, 120, and 180 min of incubation at 42°C in MHB under microaerobic conditions. The results showed various adhesion capabilities among the isolates of *C. jejuni* (**Figure 1**). In general, all *C. jejuni* isolates were able to adhere to the surface within 30 min, with the number of cells increasing with the time of incubation. When speaking about the particular isolates, the strongest adhesion ability was observed in the surface water isolate Сj5653P at 120 and 180 min (p < 0.05), while the lowest adhesion was observed in the surface water isolate Сj5683P at 120 and 180 min (p < 0.05). Statistical analysis (Spearman’s rank correlation test) showed a positive correlation adhesion capacity and time of incubation in 7 out of 15 isolates (p < 0.05, **Supplementary Table 1**).

### Architecture and Quantification of Biofilms

**Figure 2:**
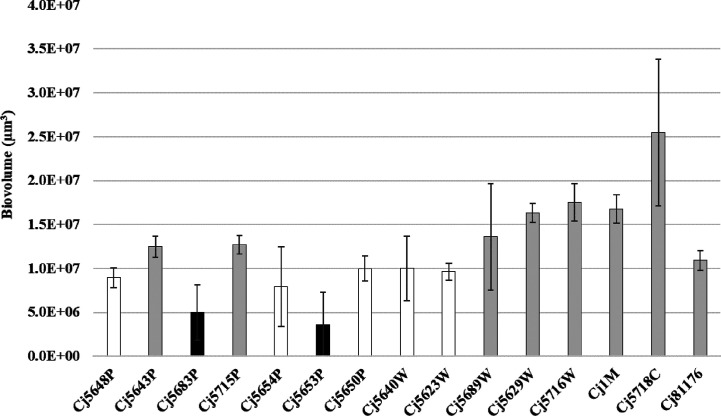
Biofilm biomass quantified by CLSM after the Syto-9 staining. Experiments were performed in triplicate of three independent cultures, the error bars represent standard deviation from the mean. Bar of the same color (white, gray and black) indicates statistically similar values (p < 0.05).

The 15 C*. jejuni* isolates were investigated for static biofilm formation with the selected cultivation protocol. According to the evaluation of CLSM images, all isolates of *C. jejuni* were able to form three-dimensional structures after 24 h of incubation. However, the quantity and the architecture of the biofilms of the isolates were diverse (**Figure 2**, **Supplementary Table 2**).

**Figure 3:**
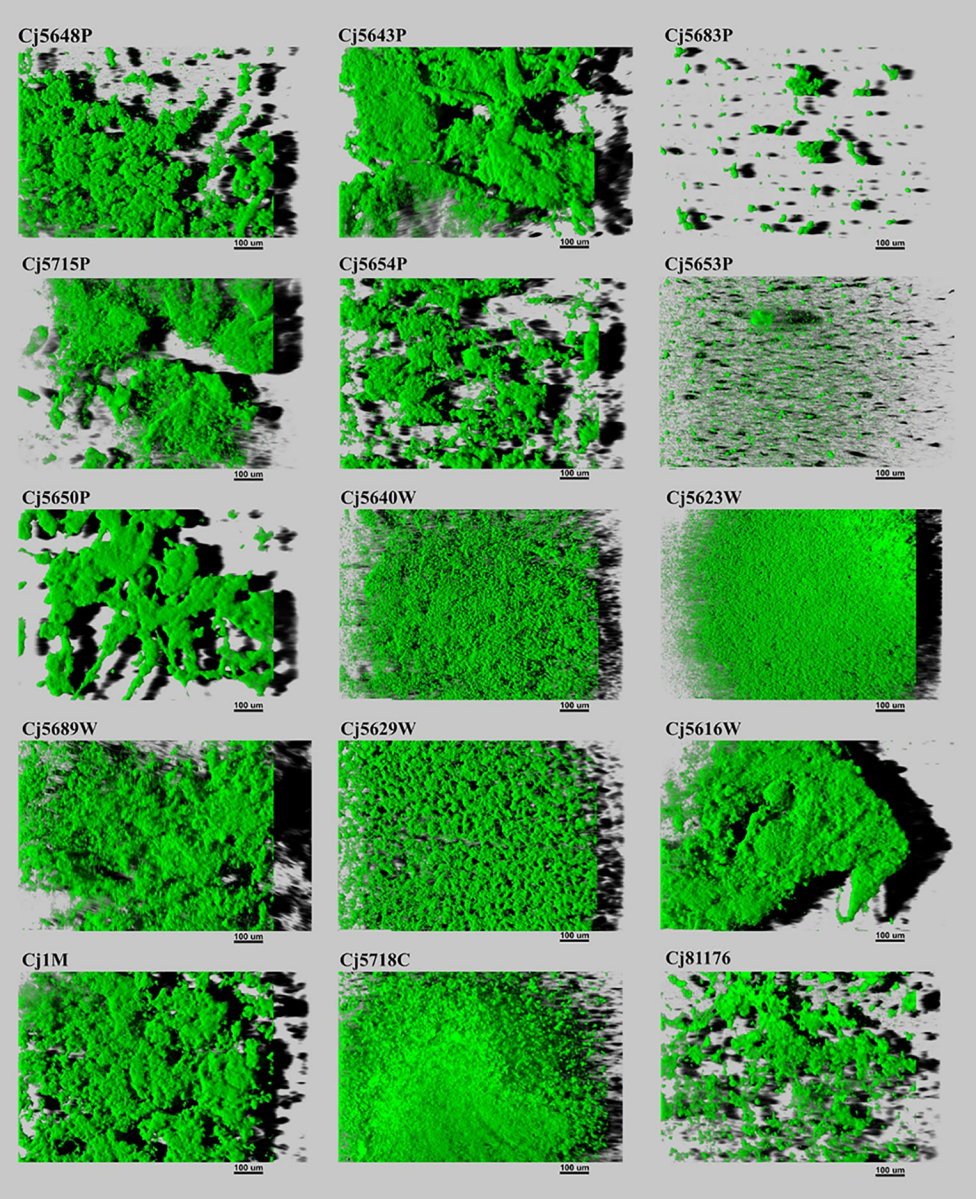
Three-dimensional projections of structures of biofilms obtained from scanning along the z-axis acquired through CLSM. The scale bar represents 100 μm. The CLSM images represent an overhead view of the biofilms formed by 15 isolates of *C. jejuni*, with virtual shadow projection to the right.

The biofilm architecture ranged from a flat homogeneous layer of cells to complex clustered structures containing hollow voids. Biofilms formed by isolates from pond water displayed as highly structured massive compact clusters. On the contrary, biofilms formed by isolates from waste water had a more homogeneous and continuous structure. Within the collection, only two surface water isolates (Cj5653P and Cj5683W) formed flat biofilms, looking like simple clusters of cells (**Figure 3**). But in general, the visual structure of the water isolates did not differ from the structure of the clinical, food, and collection strains.

When speaking about the biofilm quantity, the average amount of biovolume ranged from 3.59 × 10^6^ µm^3^ to 17.50 × 10^6^ µm^3^ in isolates isolated from different sources of water, 16.79 × 10^6^ µm^3^ in the food isolate and 10.92 × 10^6^ µm^3^ in the collection strain. The larger biovolume was produced by the clinical isolate (25.48 × 10^6^ µm^3^). The weakest ability to form biofilms was observed in two water isolates: Cj5653P and Cj5683P (3.59 × 10^6^ µm^3^ and 4.99 × 10^6^ µm^3^), even though the water isolate Cj5653P had the strongest adhesive ability. Correlation analysis of adhesion capacity and biofilm quantity revealed strong positive relationship only in one isolate (Cj5689W) after 30, 120 and 180 min of incubation. In other cases, mostly no correlation was observed. However, six isolates showed negative correlation between the adhesion and biovolume at one timepoint (**Supplementary Table 3**).

### Screening and Sequencing of the *luxS* Gene

Since the presence or absence of the *luxS* gene in the genome of *C. jejuni* may affect the ability to form a biofilm, the isolates were tested for a presence of the gene *luxS*, which is responsible for production of the communication molecules AI-2. PCR with the first specific primer set 1 (**Table 2**), which forms a characteristic 222 bp product in the inner region of the *luxS* gene, showed no amplified product in isolates Cj5653P and Cj5683P (**Supplementary Figure 1A**). The results were confirmed by second PCR with primer set 2 (**Table 2**), which bounds to the outer region around the *luxS* and forms characteristic 800 bp product (**Supplementary Figure 1B**). The results of Sanger sequencing of the *luxS-*containing fragments of positive samples showed high rate of similarity among the nucleotide sequences, reaching 95.15% to 99.79% of homology with the collection strain 81 to 176 (**Supplementary Figure 2**). Translation of nucleotide sequence to the amino acid sequence showed several differences (**Table 3**), which could be important for the future studies of the function of the *luxS* gene and the ability to produce the signal molecule. Sequencing data is available in the NCBI database under the following numbers: MT432260, MT432261, MT432262, MT432263, MT432264, MT432265, MT432266, MT432267, MT432268, MT432269, MT432270, MT432271.

**Table 3:** Differences in LuxS amino acid sequences of isolates as compared to the collection strain *C. jejuni* NCTC 11168.

Isolate	Amino acid variation and their position*
Cj5718C	I→V (100); A→E (106); I→M (154)
Cj5650P	I→V (100); A→E (106); I→M (154)
Cj5689W	I→V (100) A→E (106); I→M (154)
Cj5640W	I→V (100); E→K (105); I→M (154)
Cj5715P	I→V (42, 100); N→D (71); I→M (154); A→E (106)
Cj5716W	L→F (161); I→M (154); A→E (106)
Cj81176	D→N (10); I→V (42); N→D (71); I→V (100); A→E (106); I→M (154)
Cj5648P	A→E (106); I→M (154)
Cj5654P	A→E (106); I→M (154)
Cj5643P	Same as NCTC 11168
Cj5623W	Same as NCTC 11168
Cj5629W	Same as NCTC 11168
Cj1M	Same as NCTC 11168
Cj5653P	NA
Cj5683P	NA

*A, alanine; D, aspartate; E, glutamate; F, phenylalanine; I, isoleucine; K, lysine; L, leucine; M, methionine; N, asparagine; V, valine.

## Discussion

Due to the high prevalence of human infections caused by *C. jejuni* throughout the world, it is important to understand the ability of this pathogen to persist in the environment and understand the risks it represents to the public health. One of the critical factors ensuring its protection against harsh conditions is its ability to form biofilms (Donlan and Costerton, 2002; Chmielewski and Frank, 2003; Hall-Stoodley et al., 2004).

Although numerous studies have shown that *C. jejuni* can form biofilms on abiotic surfaces, there is very little information concerning environmental isolates, in particular those isolated from water, even though they represent a potential source of infection (Hänninen et al., 2003; Mughini-Gras et al., 2006; Sparks, 2019). Therefore, this work was focused on a comparison of the adhesion and biofilm formation ability of isolates of different origin (surface and waste water, food, and clinical isolates). All tested isolates were able to adhere to the microtiter plate within the first 30 min of incubation, although the numbers of attached cells differed among the isolates. Similar diversity was observed when comparing the volume of the subsequently produced biofilms. Interestingly, even though the adhesion is the first and crucial step of the biofilm formation process, it seems that the adhesion capacity is not directly proportional to the level of the biofilm formation ability, as only one isolate showed positive correlation between the adhesion capacity and the quantity of the biofilm. Moreover, negative correlation was observed in six isolates. However, it is important to mention, that the biofilms were quantified after 24 h of incubation and could, therefore, be in their dispersal phase. To reveal the true relation between the adhesion capacity and biofilm formation ability, further experiments involving measurements of biofilm formation dynamics are needed.

Leaving aside the quantitative analysis, the architecture of the biofilms formed by the tested isolates was also very diverse. The structure of *C. jejuni* biofilms can vary from a monolayer of adherent cells, through flat unstructured multilayers, up to highly structured biomass of clusters containing water channels and voids (Turonova et al., 2015; Bronnec et al., 2016). The architecture of the biofilm mostly depends on the strains used for the experiments and on the experimental design (Buswell et al., 1998; Joshua et al., 2006). Interestingly, the isolates obtained from the wastewater treatment plant formed more compact biofilms than the isolates from other origins. Their structure was almost carpet-like, with various numbers of small channels and voids. This architecture could be related to the stress that *C. jejuni* cells encounter during the treatment of the water - compact structure with fewer voids means that only a small proportion of the cells is exposed to the chemicals used for the water treatment. Moreover, the affected cells can pass the information about the present danger to the deeper layers of the biofilm *via* quorum sensing, giving the remaining cells enough time to adjust and therefore to ensure the survival of the population.

It is well known that quorum sensing, also known as cell-to-cell signaling, plays a role in biofilm formation (Plummer, 2012). Previous studies reported that *C. jejuni* strains that lack the *luxS* gene responsible for the production of autoinducer-2 molecules had a reduced ability to form static biofilms (Reeser et al., 2007). According to some sources, the distribution of the *luxS* gene within the genus of *C. jejuni* is not uniform and is often missing, especially in environmental isolates (Hepworth et al., 2011). However, in this work *luxS* was absent in only two out of the 12 tested water isolates. The sequence of the gene was relatively well conserved, although the translation to the amino acid sequence revealed several variations, mostly alanine instead of glutamate at position 106, and valine instead of isoleucine at position 100. Interestingly, the strains containing the respective substitutions did not show any similarities in adhesion capacity, biofilm quantity, or the biofilm architecture. Therefore, either the substitution of the amino acids does not cause a functional change of the LuxS enzyme, or it does not influence these particular characteristics of the biofilm formation. Recently, Plummer et al. (2011) described a mutation G92D in *C. jejuni* 81116, which resulted in the loss of production of the AI-2 as well as in decreased the catalytic activity of LuxS in comparison to the wild-type. However, this type of mutation was not detected in among the inspected strains.

The two isolates with missing *luxS* were isolated from surface water (pond) and both produced thin sparse biofilms, lacking the presence of significant clusters (complex interconnected parts of the biomass). Despite the absent *luxS* and reduced biofilm formation ability, the isolates were able to adhere to the surface. Moreover, one of them was marked as the isolate with the highest adhesion ability. These results suggest that the presence or absence of the *luxS* gene itself may have a decisive effect on biofilm formation and clustering ability, but does not affect the adhesion itself. This is in contrast with the data published by Quiñones et al. (2009), who observed reduced adhesion ability in mutants lacking the *luxS* gene. However, the authors used different methodology to assess adhesion capacity.

Overall, this work showed that water isolates of *C. jejuni* can adhere to a surface and subsequently form a spatially structured biofilm. As their adhesion capacity was comparable to the strains of clinical or food origin, they might indeed represent a significant source of contamination in animal husbandry, and as a source of infection in humans. However, further research is needed to evaluate their virulence and persistence in the environment.

## Data Availability Statement

The original contributions presented in the study are publicly available. This data can be found here: https://www.ncbi.nlm.nih.gov/ MT432260, MT432261, MT432262, MT432263, MT432264, MT432265, MT432266, MT432267, MT432268, MT432269, MT432270, MT432271.

## Author Contributions

HM, RK, and KD conceived the project. ES and NS provided *C. jejuni* isolates from surface and waste water. ES, MT, and HM designed the experiments, and analyzed and interpreted the data. ES and MT performed the experiments. ES wrote the manuscript. All authors contributed to the article and approved the submitted version.

## Funding

This research was supported by the The Czech Science Foundation (No. GA18-16549S).

## Conflict of Interest

The authors declare that the research was conducted in the absence of any commercial or financial relationships that could be construed as a potential conflict of interest.
