# Supplementary material for: Adhesion, Biofilm Formation, and luxS Sequencing of Campylobacter jejuni Isolated From Water in the Czech Republic
Source: Front Cell Infect Microbiol. 2020 Nov 16;10:596613. doi: 10.3389/fcimb.2020.596613 (PMC7718015; doi:10.3389/fcimb.2020.596613)
Supplement: Supplementary file 1 [file DataSheet_1.zip › Supplementary Material.docx]

**Supplementary table 1.** Correlation between levels of adhesion at different time of incubation of *C. jejuni* isolates.

∗p < 0.05; ∗∗p < 0.01

| *C. jejuni* isolate | Spearman's rank correlation coefficient (r) | | | | |
| --- | --- | --- | --- | --- | --- |
|  | **30 vs. 120 min** | **30 vs. 180 min** | **60 vs. 120 min** | **60 vs. 180 min** | **120 vs. 180 min** |
| Cj5683P |  |  | r =0,715* |  |  |
| Cj5650P |  |  | r =0,714* |  |  |
| Cj5640W |  |  | r =0,711* |  | r =0,764* |
| Cj5623W |  |  |  | r =0,695^*^ |  |
| Cj5689W |  | r =0,821^**^ |  |  | r =0,776* |
| Cj5629W | r =0,811** |  | r =0,683* |  |  |
| Cj5716W |  |  | r =0,782* |  |  |

**Supplementary table 2.** Biovolume of the biofilms formed by *C. jejuni*. Data represent the mean and standard deviation of at least three independent biological replicates.

| Isolate | Biovolume (µm^3^) | Standard deviation |
| --- | --- | --- |
| Cj5648P | 8.97 x 10^6^ | 1.14 x 10^6^ |
| Cj5643P | 15.50 x 10^6^ | 1.2 x 10^6^ |
| Cj5683P | 4.99 x 10^6^ | 3.15 x 10^6^ |
| Cj5715P | 12.71 x 10^6^ | 1.03 x 10^6^ |
| Cj5654P | 7.94 x 10^6^ | 4.51 x 10^6^ |
| Cj5653P | 3.59 x 10^6^ | 3.71 x 10^6^ |
| Cj5650P | 9.99 x 10^6^ | 1.41 x 10^6^ |
| Cj5640W | 10.02 x 10^6^ | 3.67 x 10^6^ |
| Cj5623W | 9.64 x 10^6^ | 0.96 x 10^6^ |
| Cj5689W | 13.61 x 10^6^ | 6.08 x 10^6^ |
| Cj5629W | 16.34 x 10^6^ | 1.12 x 10^6^ |
| Cj5716W | 17.50 x 10^6^ | 2.14 x 10^6^ |
| Cj1M | 16.79 x 10^6^ | 1.59 x 10^6^ |
| Cj5718C | 25.48 x 10^6^ | 8.35 x 10^6^ |
| Cj81176 | 10.92 x 10^6^ | 1.13 x 10^6^ |

**Supplementary table 3**. Correlation between the adhesion capacity at different timepoints and the biofilm quantity after 24 h of incubation. Isolates showing no correlation are not listed in the table.

∗p < 0.05; ∗∗p < 0.01

| *C. jejuni* isolate | Spearman's rank correlation coefficient (r) | | | |
| --- | --- | --- | --- | --- |
|  | 30 min | 60 min | 120 min | 180 min |
| Ci5648P | - | r =-0,683* | - | - |
| Cj5643P | - | r =-0,891^**^ | - | - |
| Cj5715P | r =-0,874** | - | - | - |
| Cj5654P | - | - | r =-0,874^**^ | - |
| Cj5653P | r =-0,782* | - | - | - |
| Cj5689W | r =0,820^**^ |  | r =0,824^**^ | r =0,673^*^ |
| Cj1M | - | - | - | r =-0,703^*^ |

**
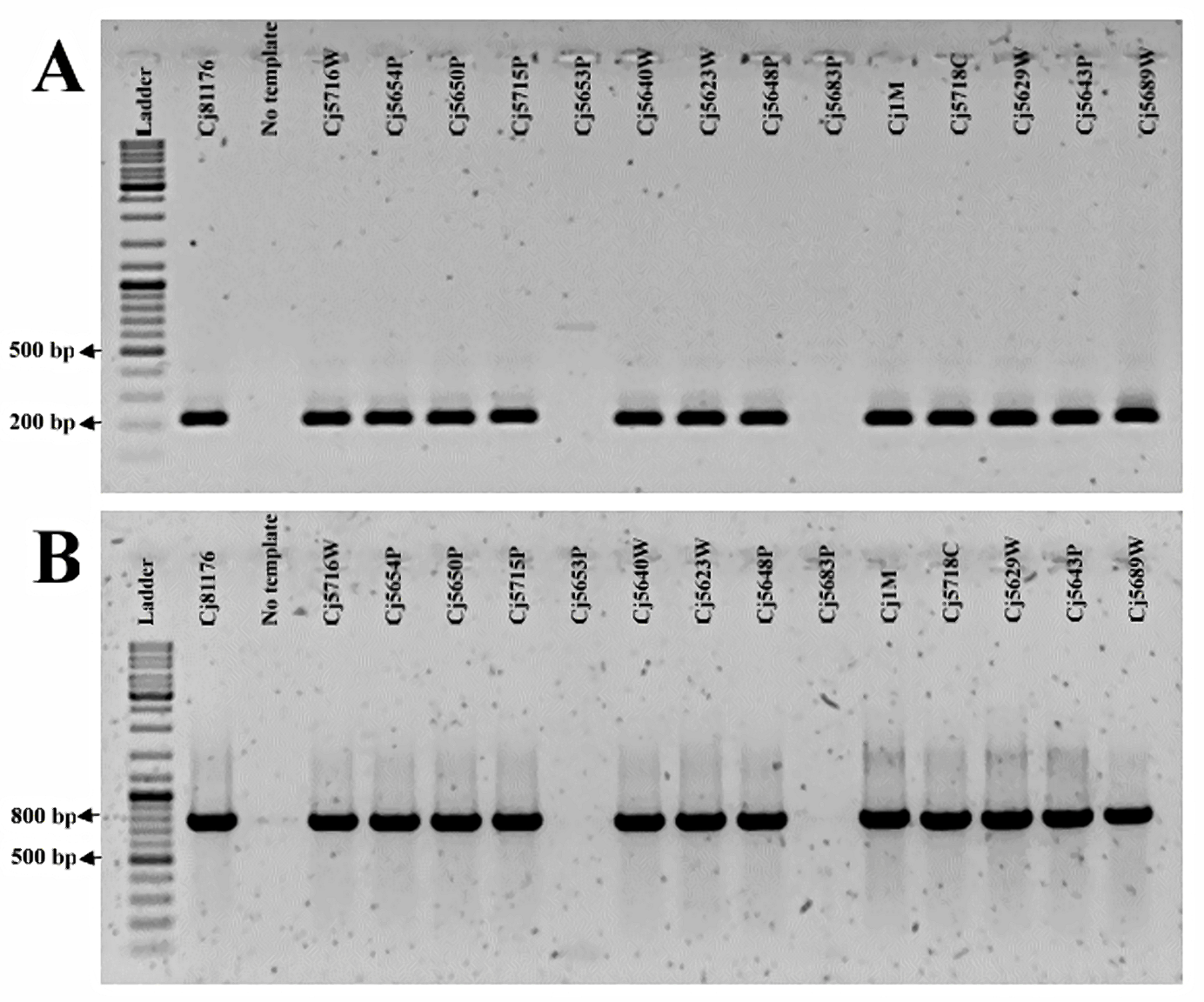
**

**Supplementary figure 1.** PCR confirmation of the presence of the *luxS* gene in tested isolates of *C. jejuni*. **A)** Bands of 222 bp product formed in the inner sequence of the *luxS* gene after using the primer set 1. **B)** Bands of 800 bp product formed in the inner sequence of the *luxS* gene after using the primer set 2. The GeneRuler 1kbp DNA Ladder (Thermo scientific, USA) was used.


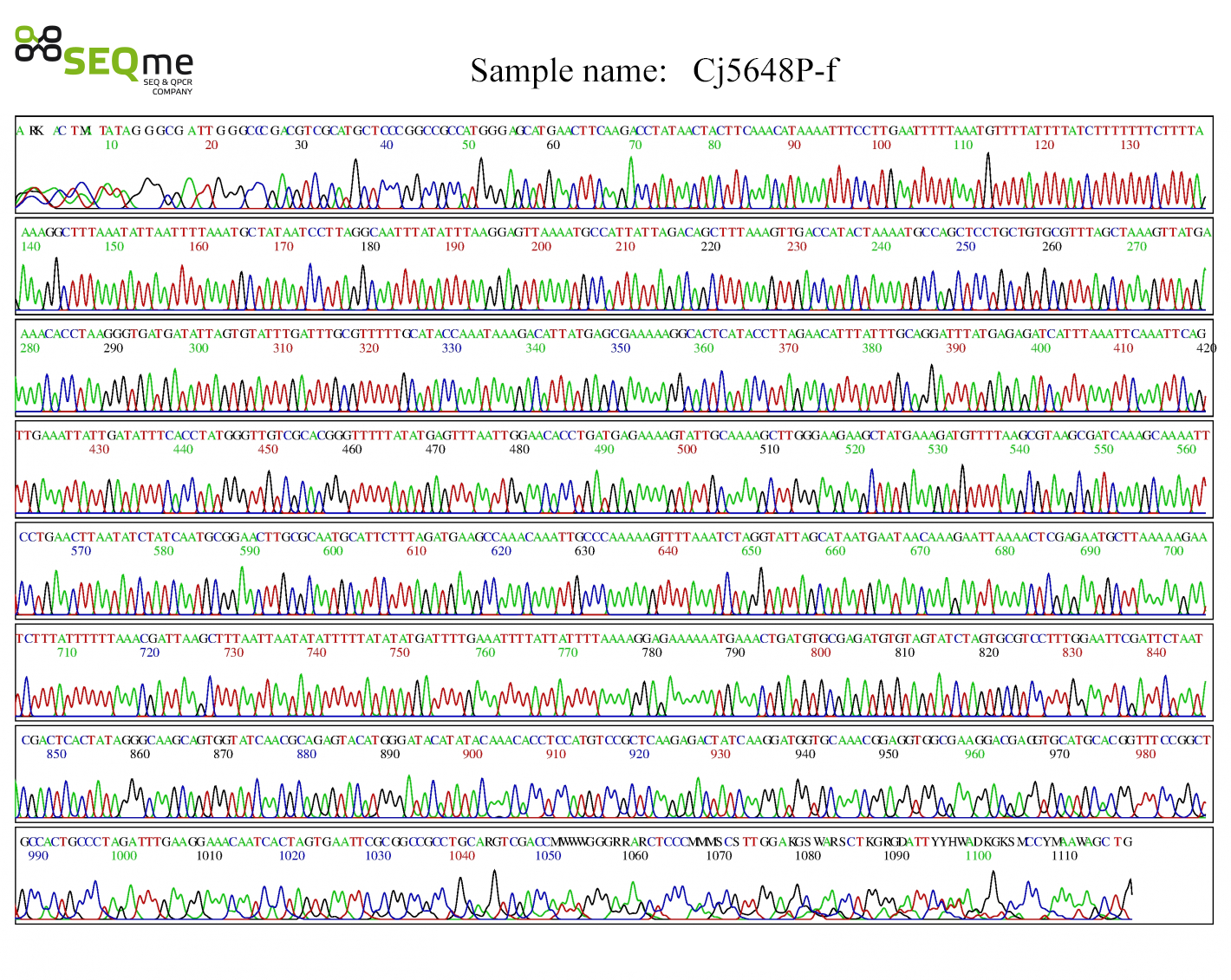

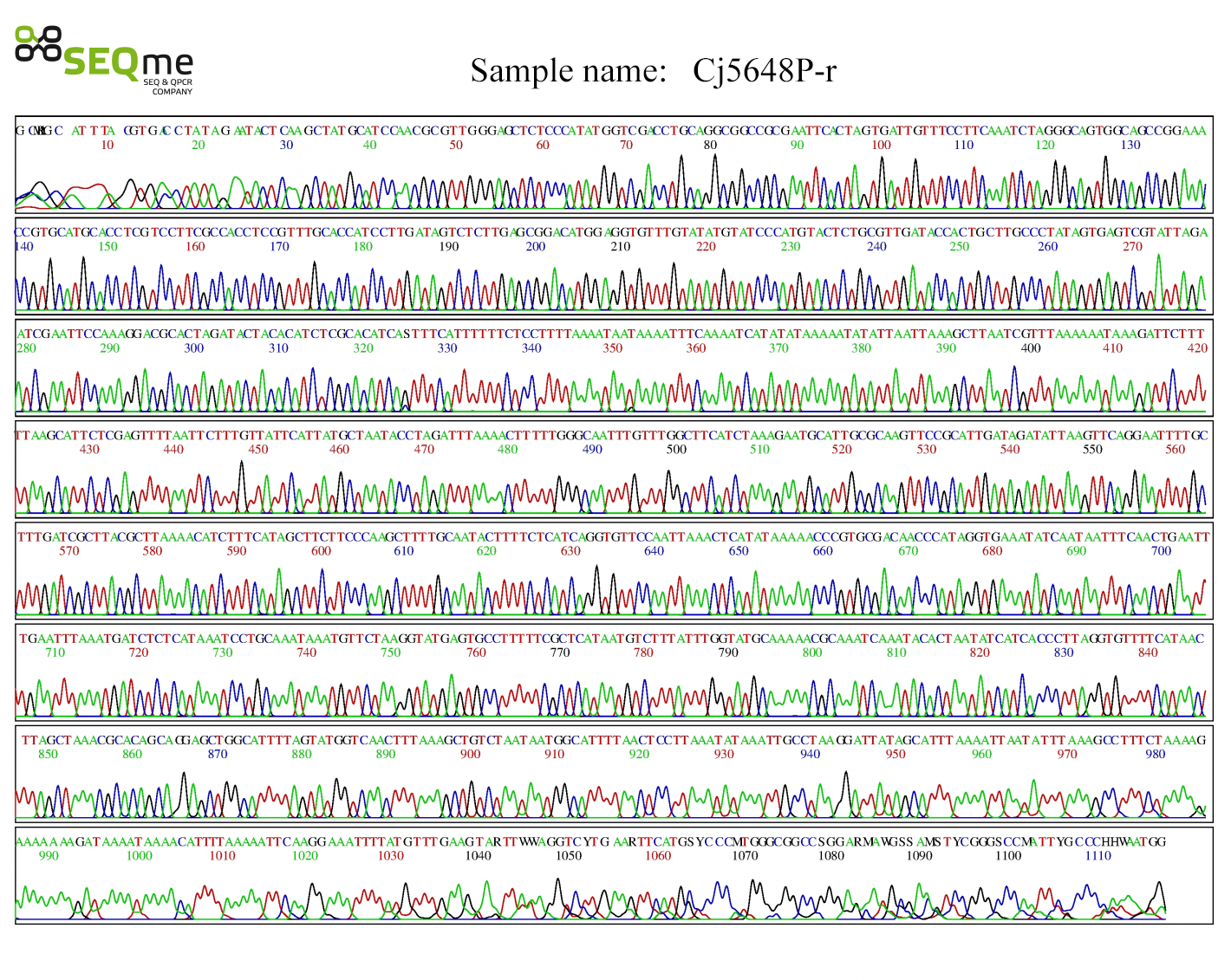

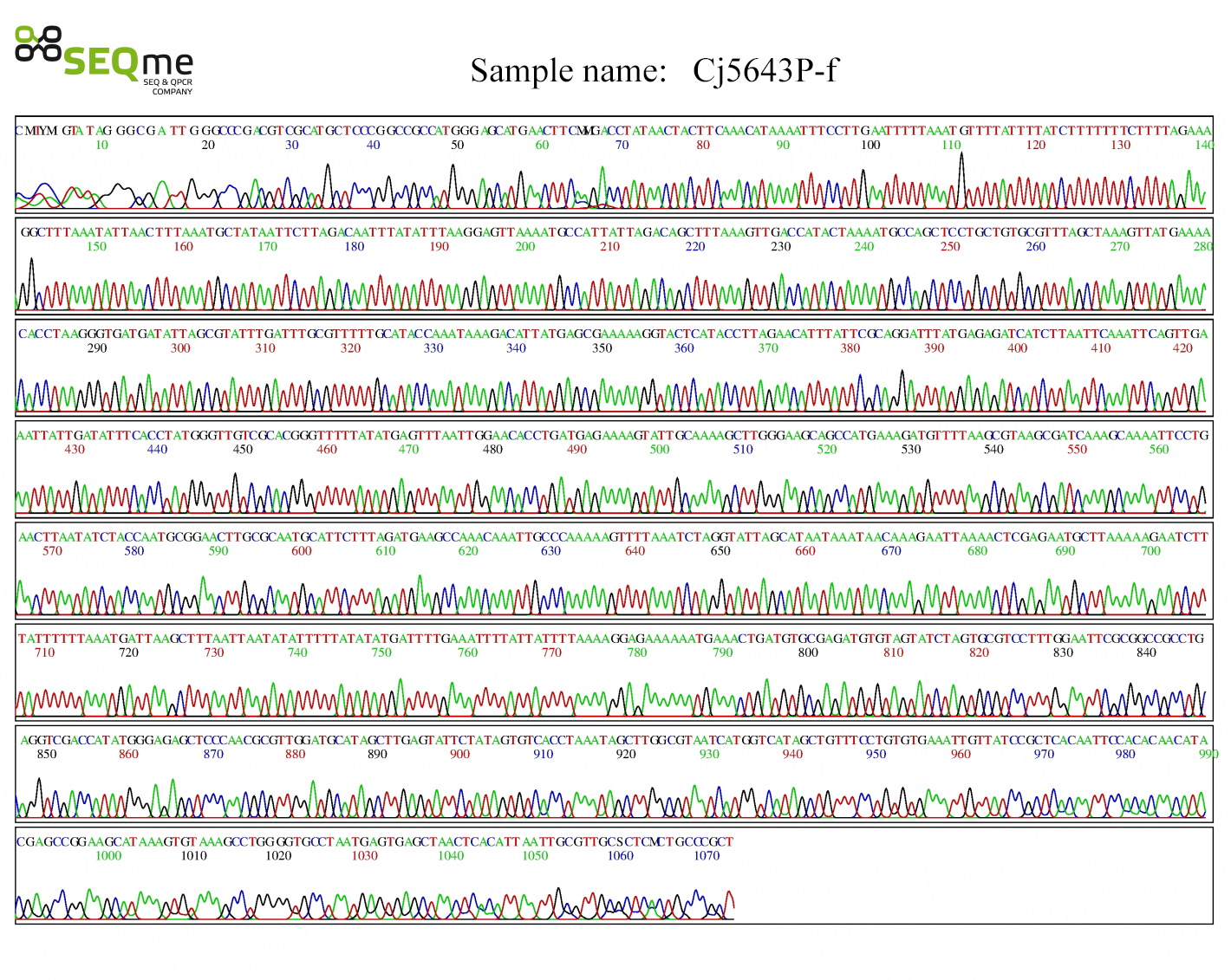

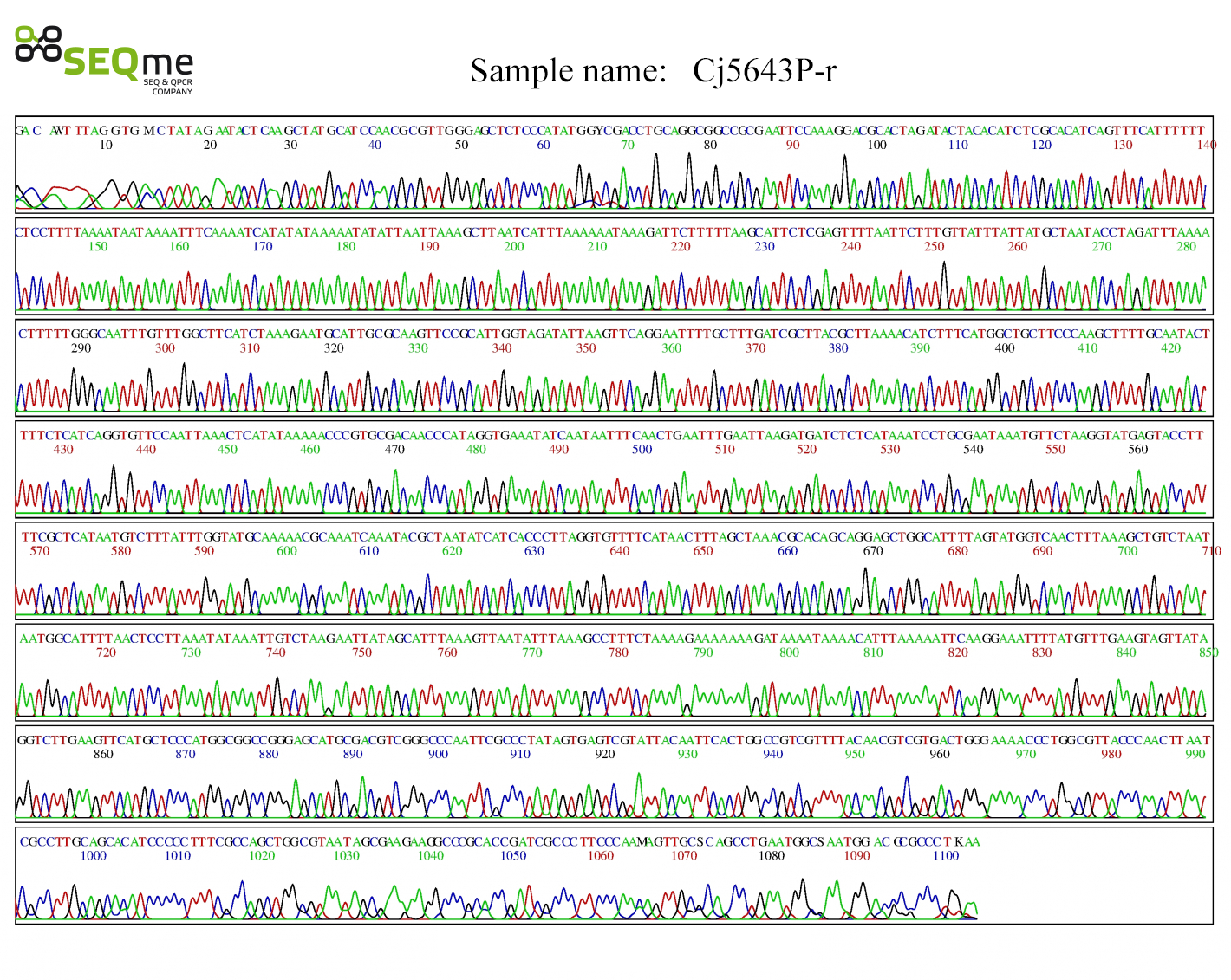

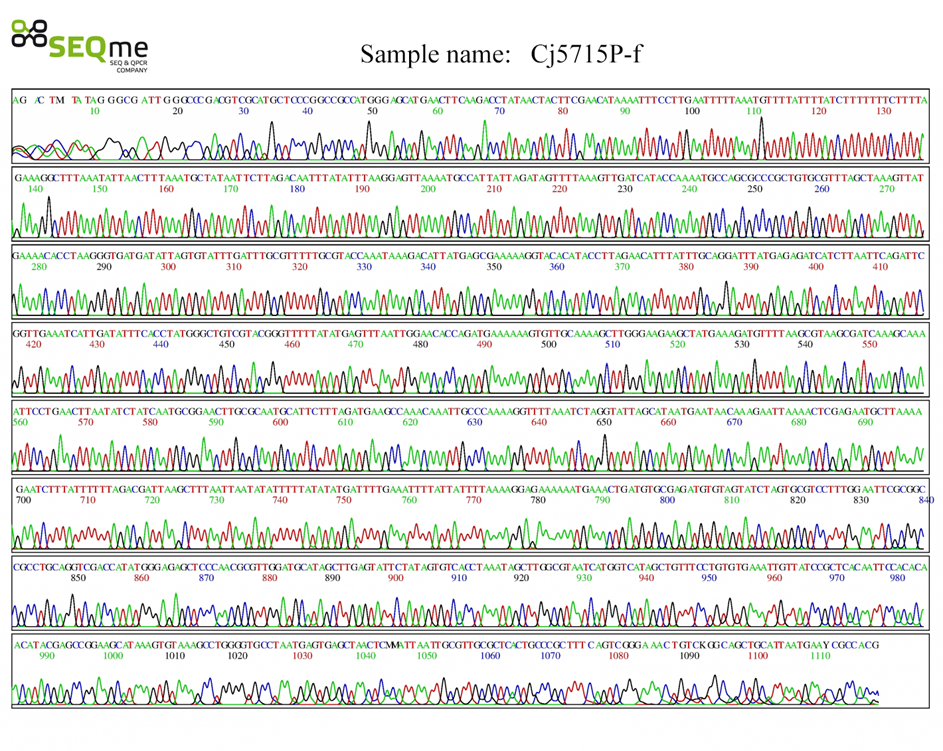


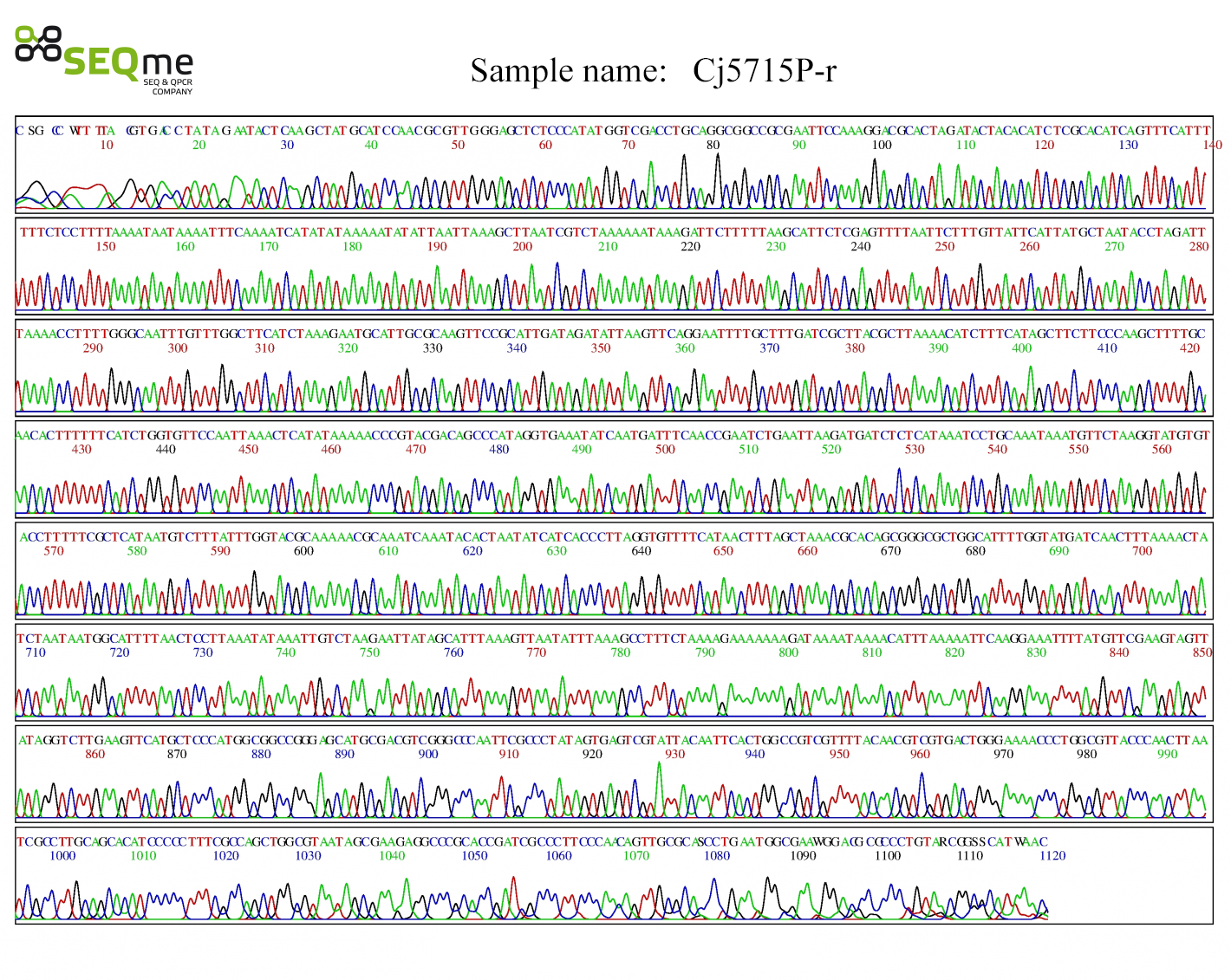

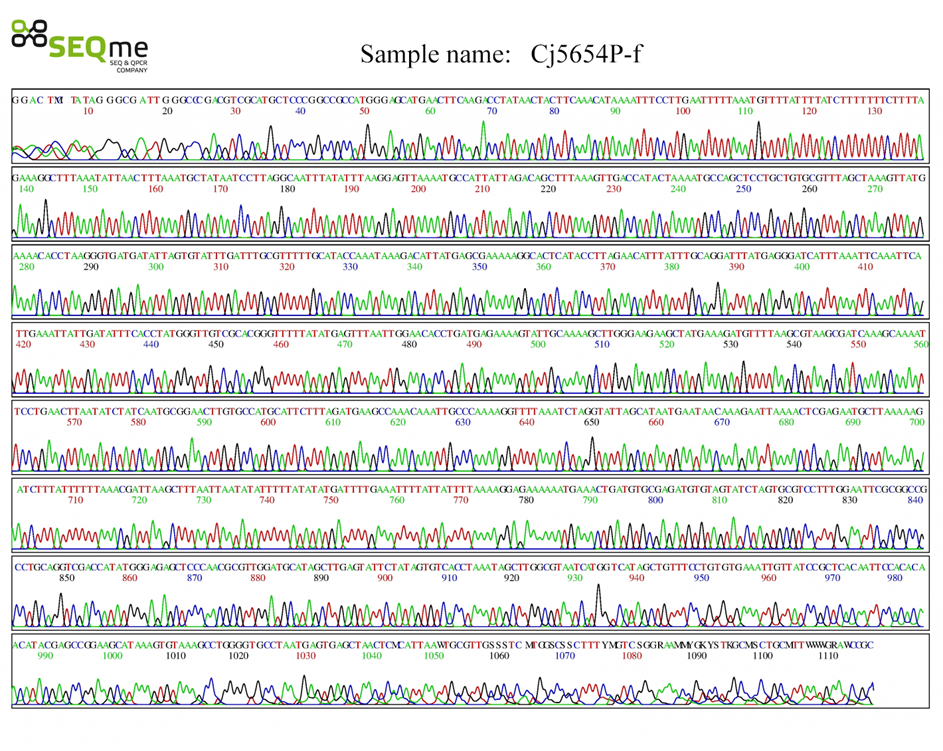


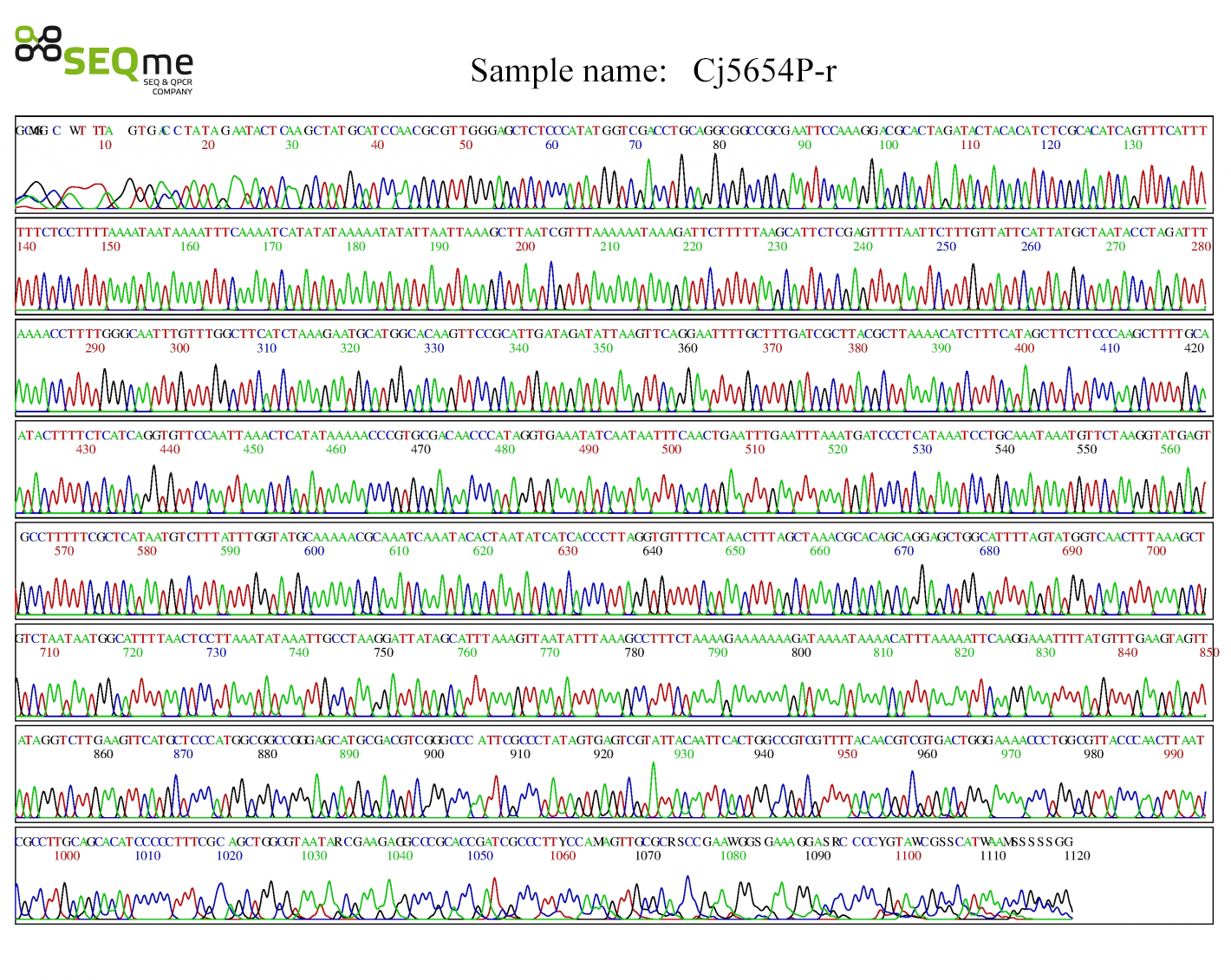

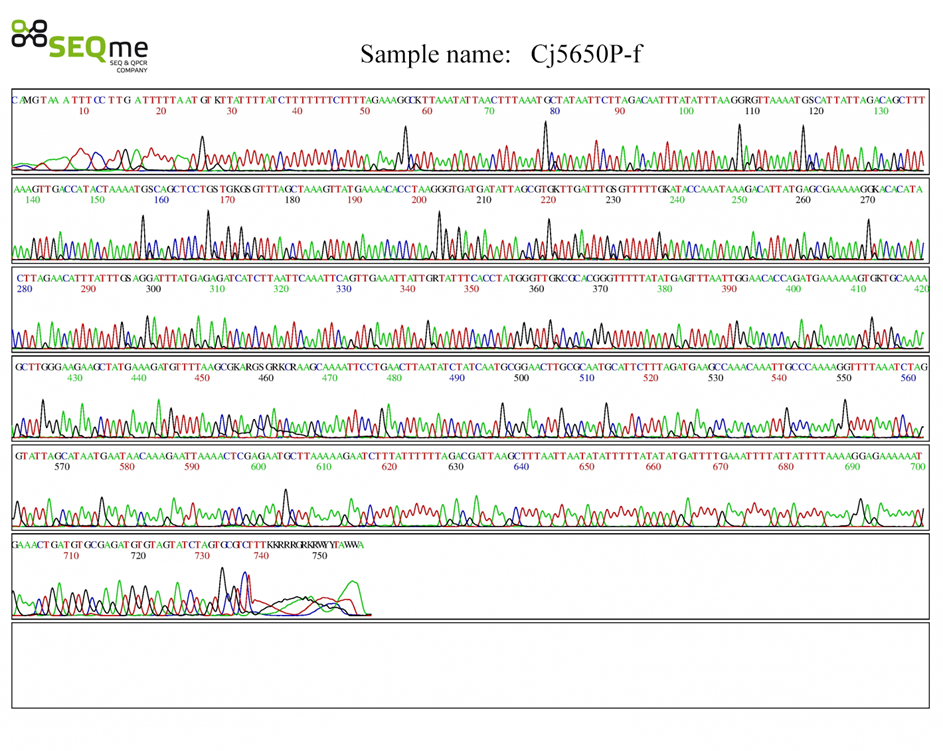


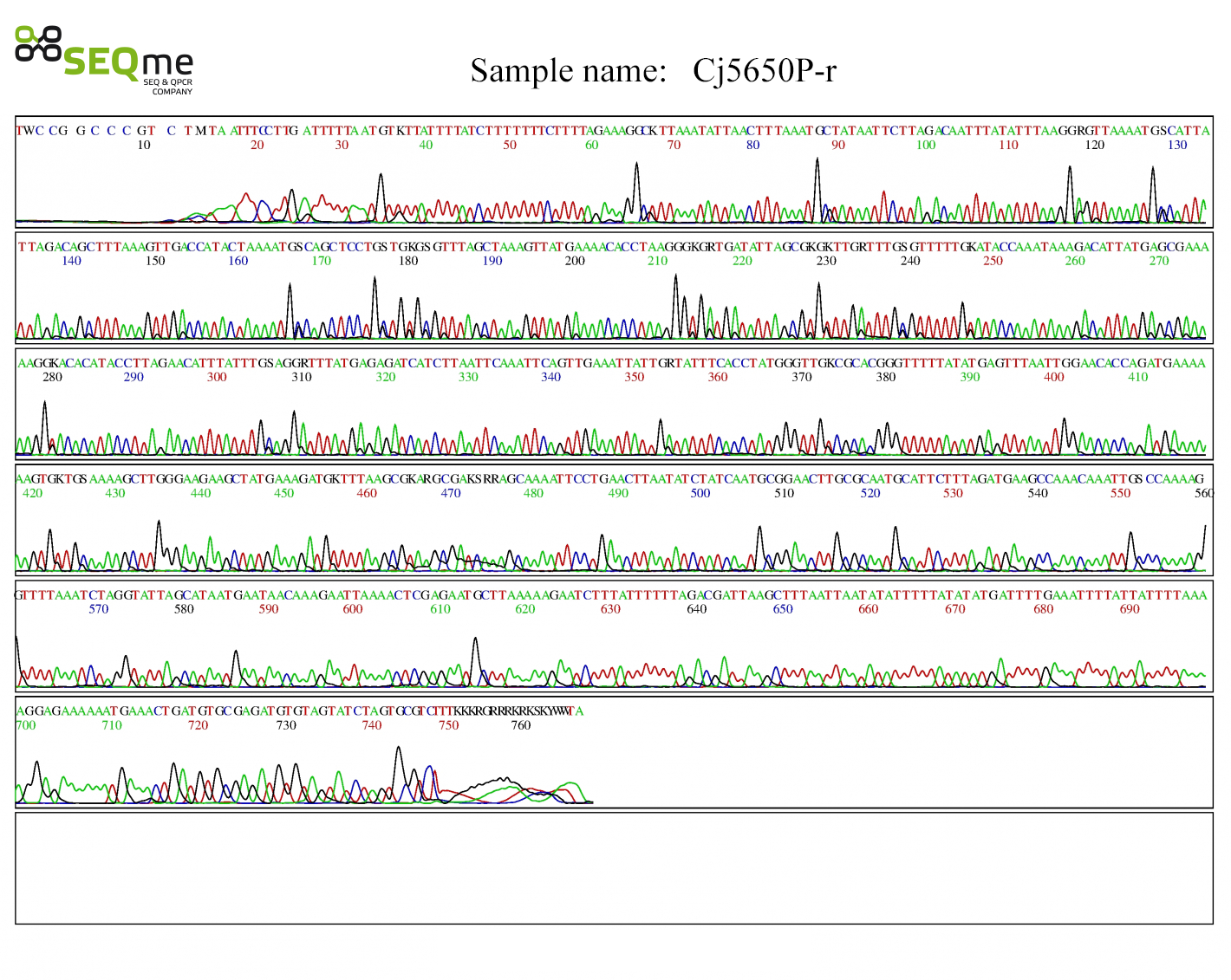

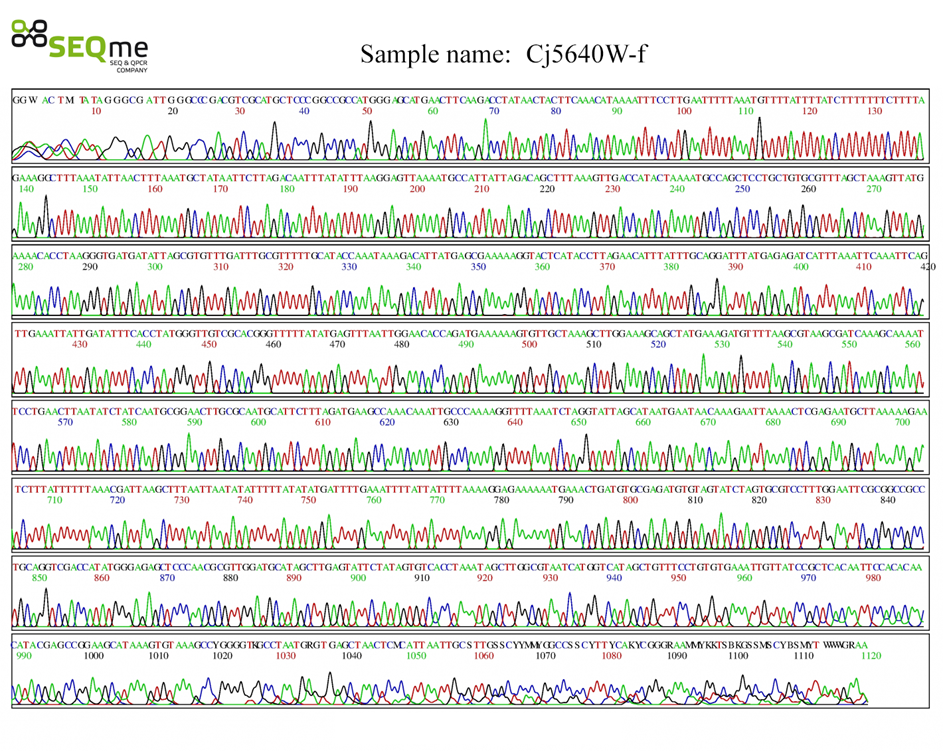


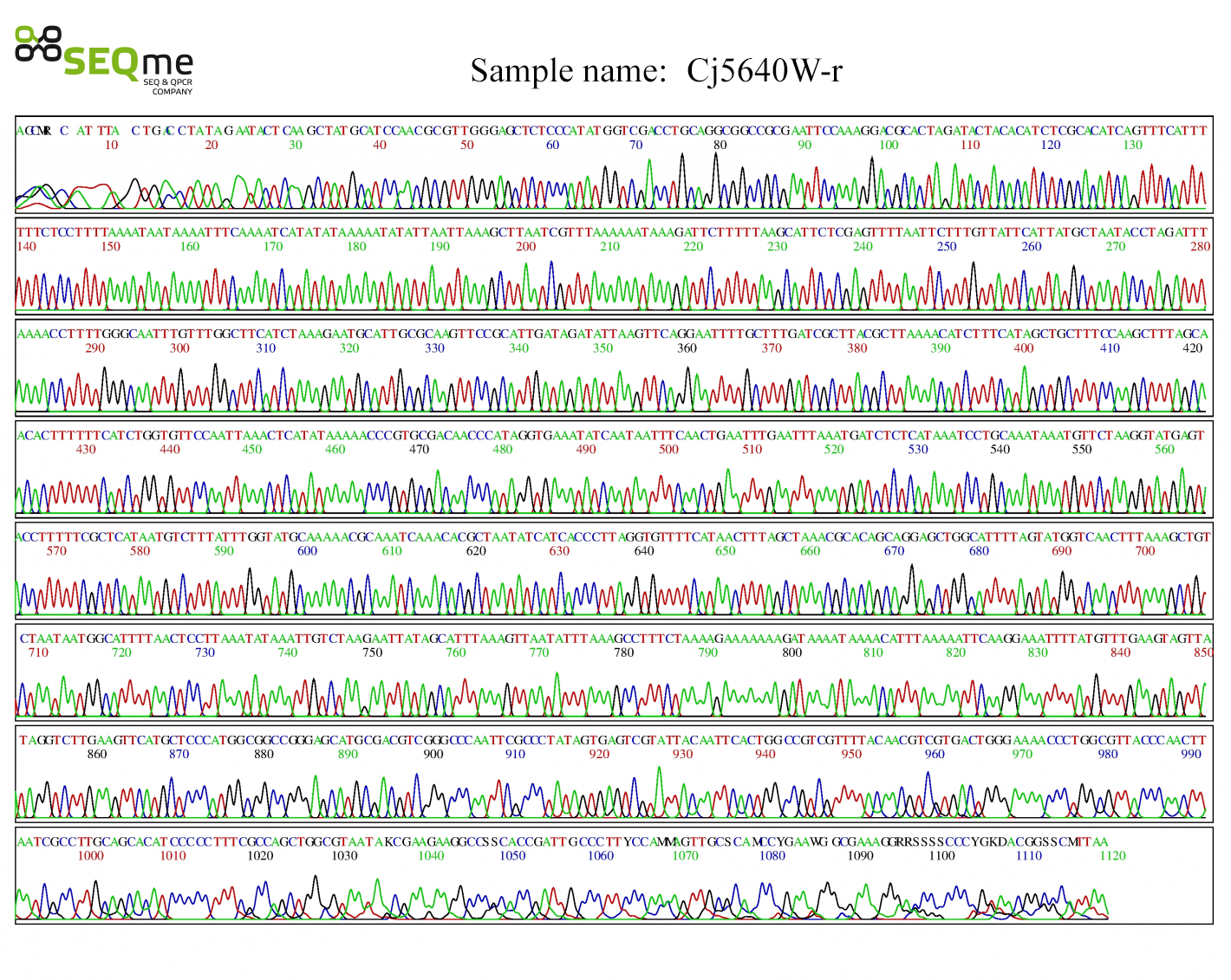

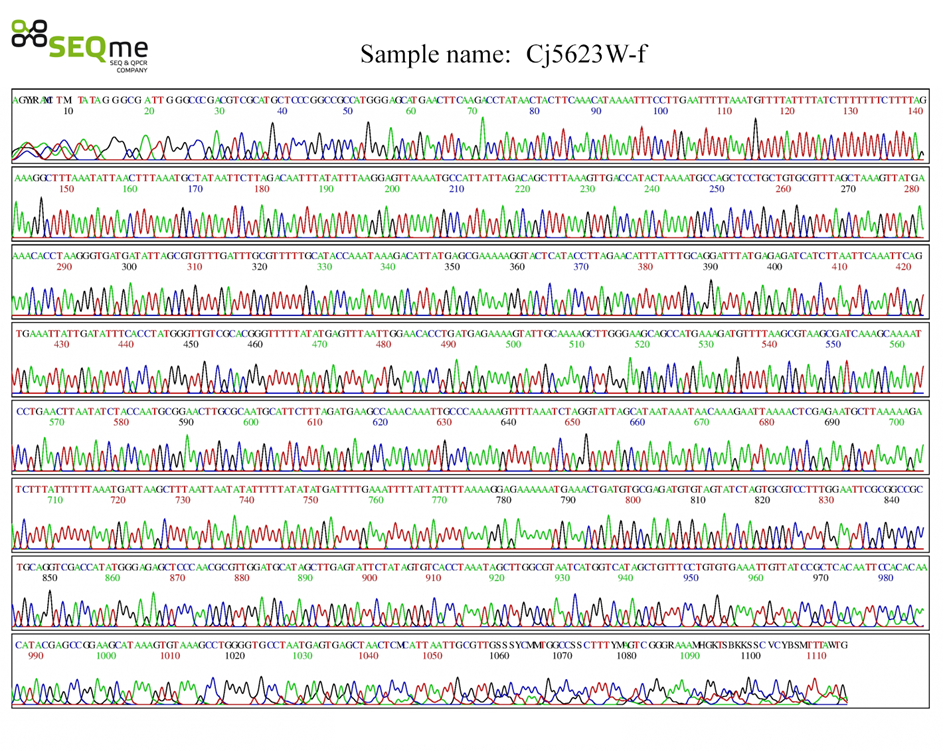


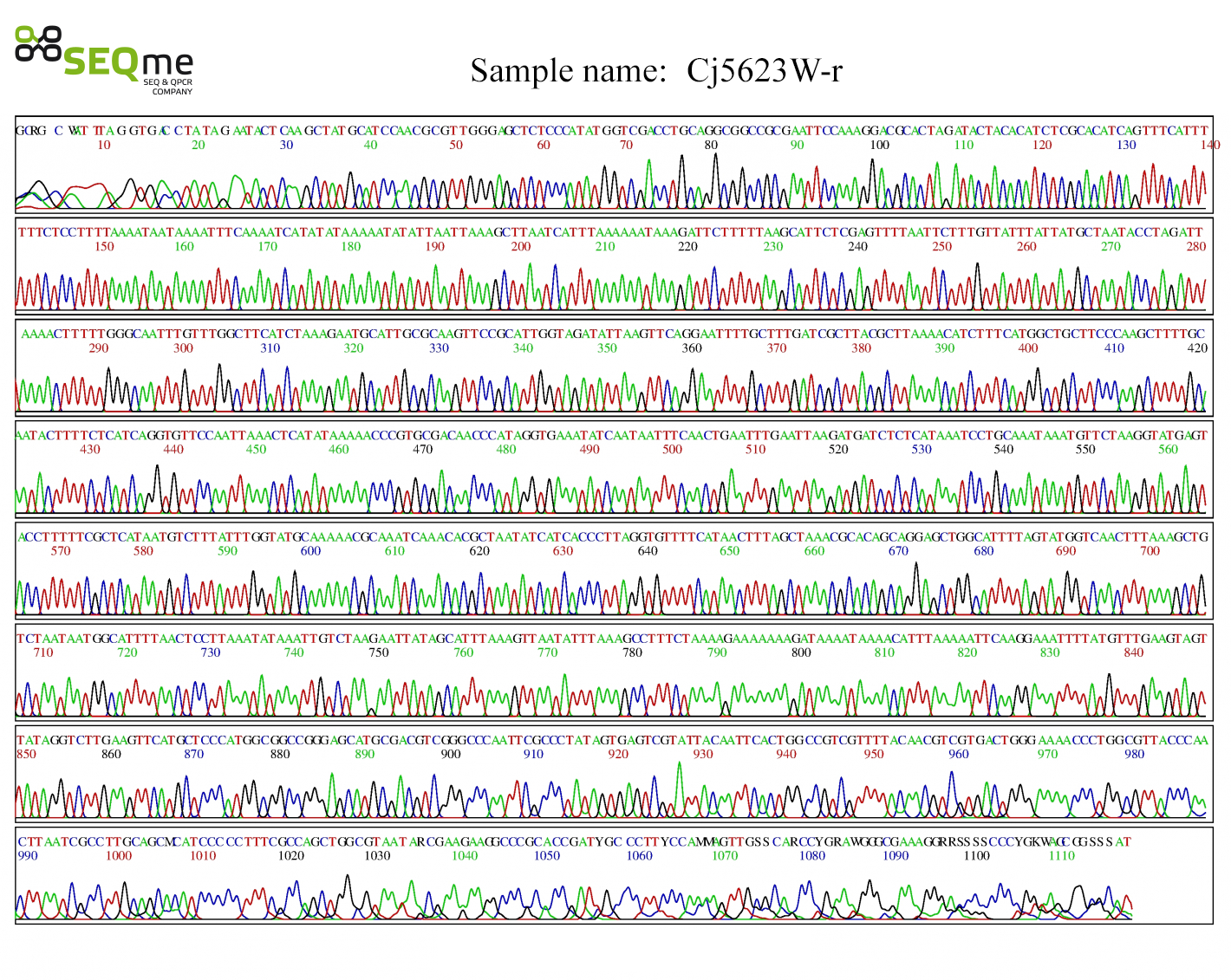

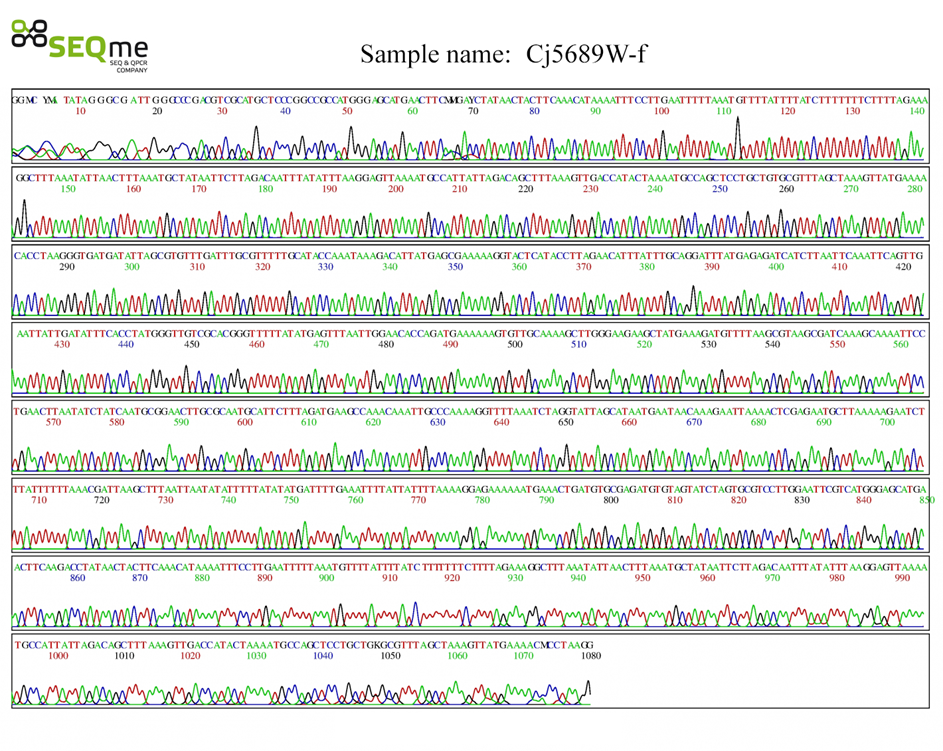


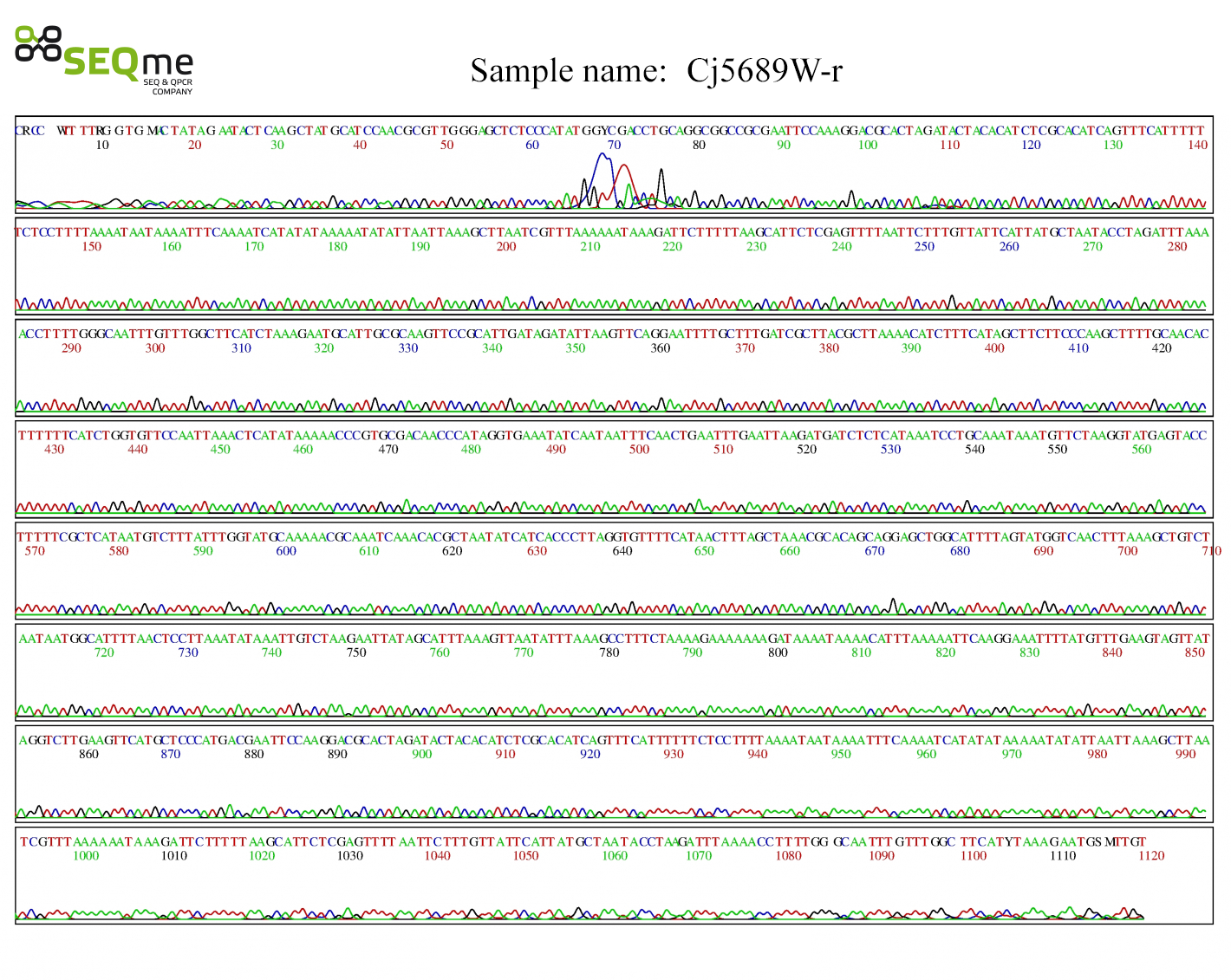

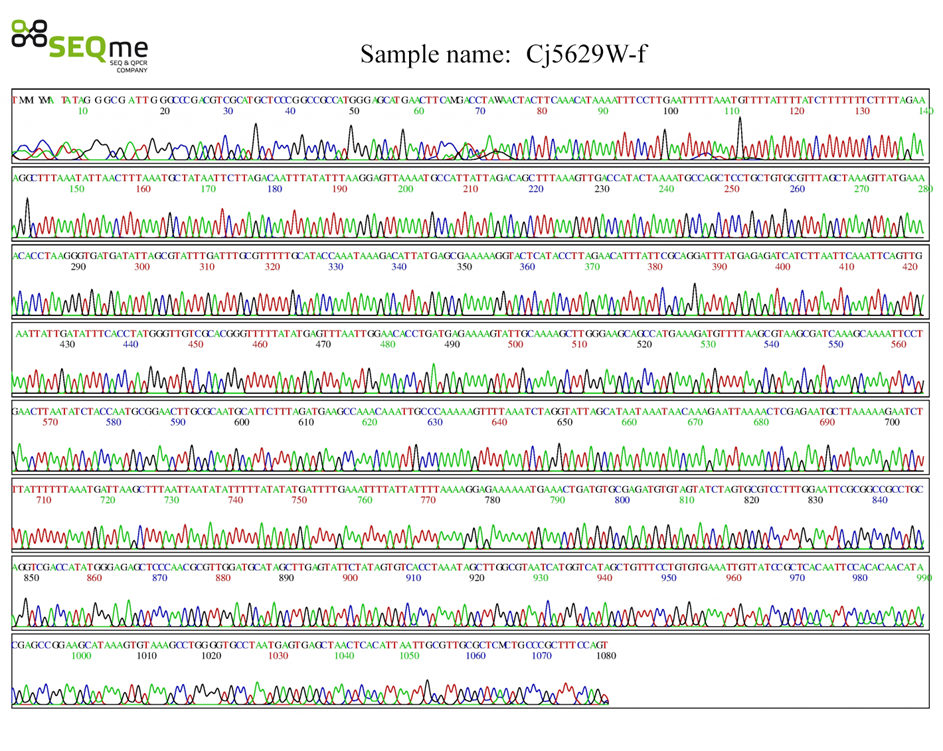


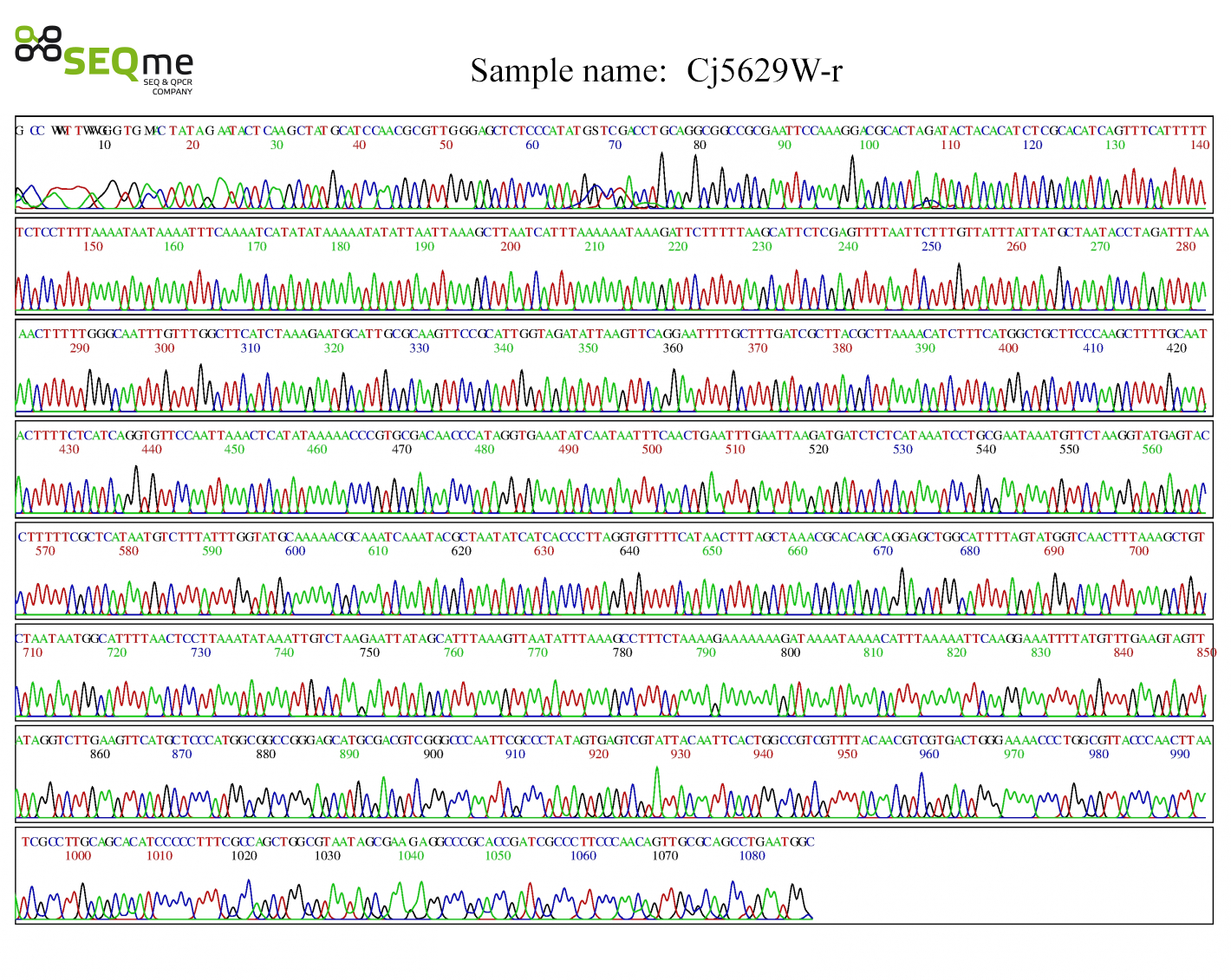

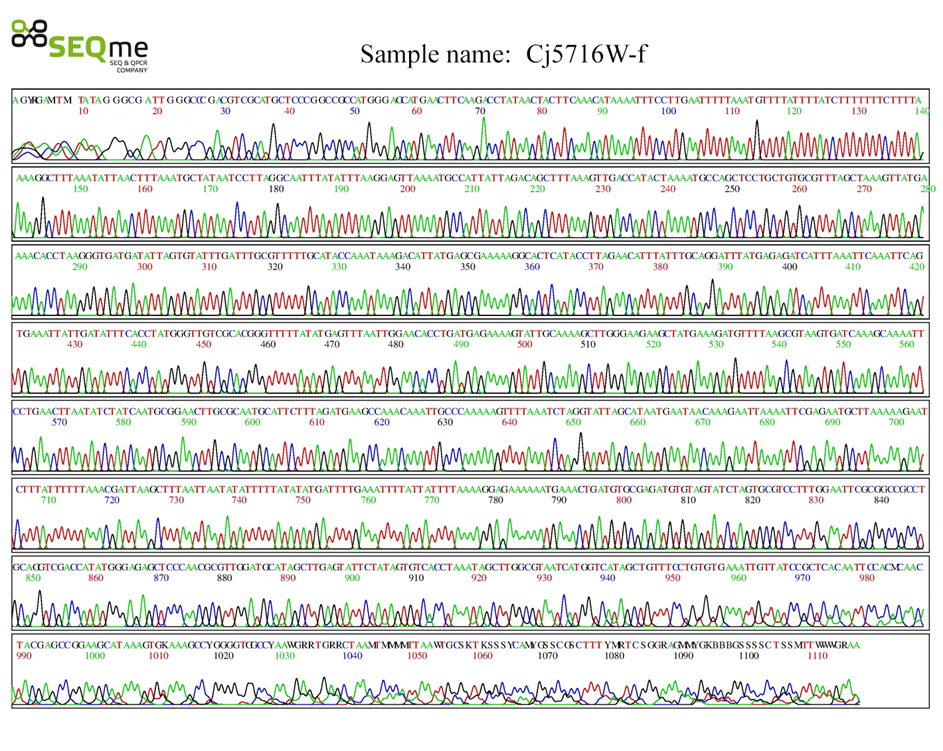


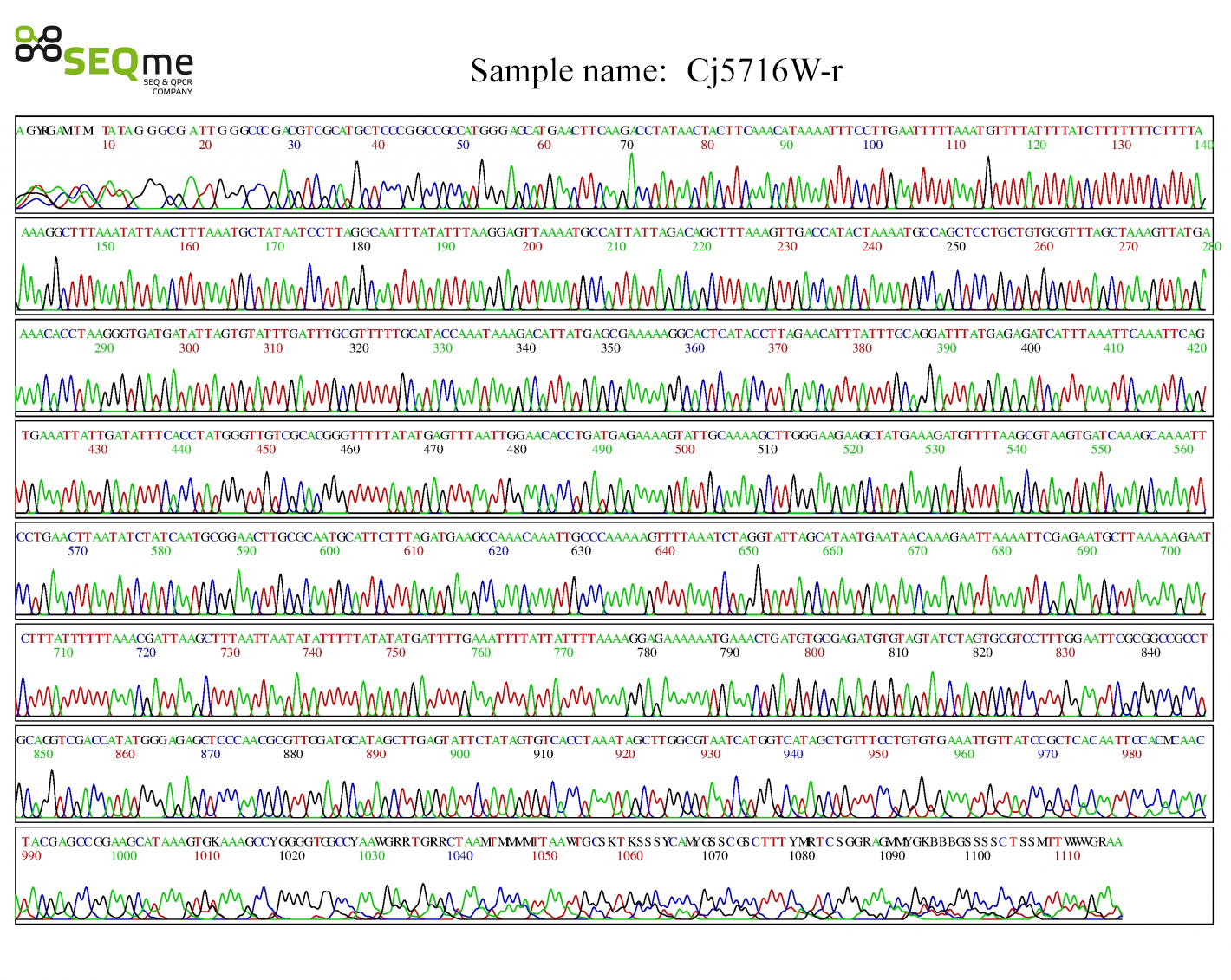

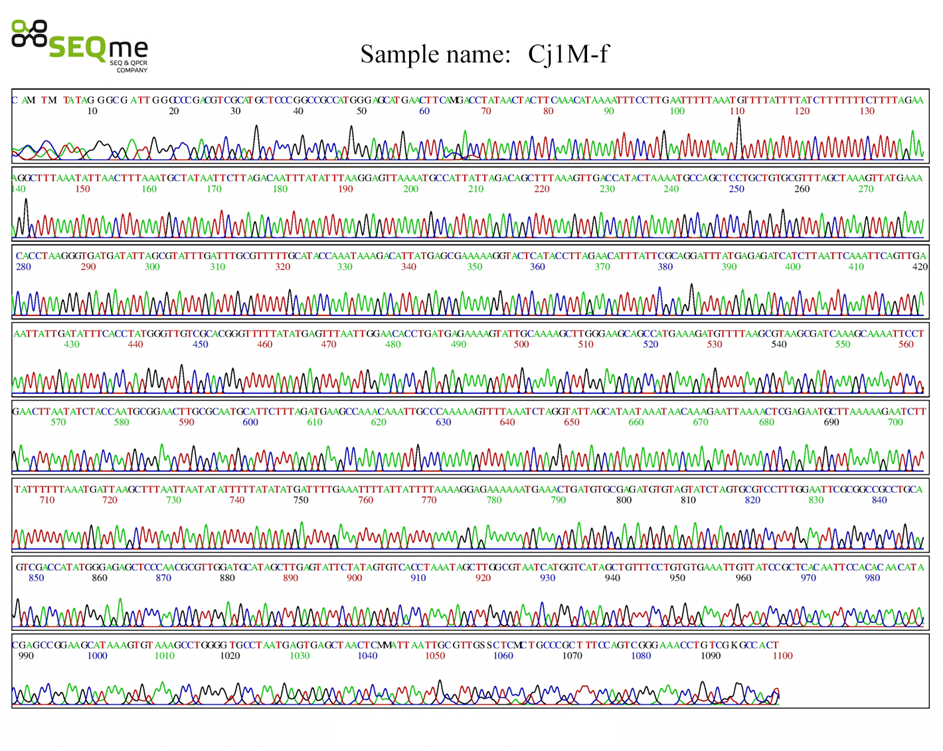


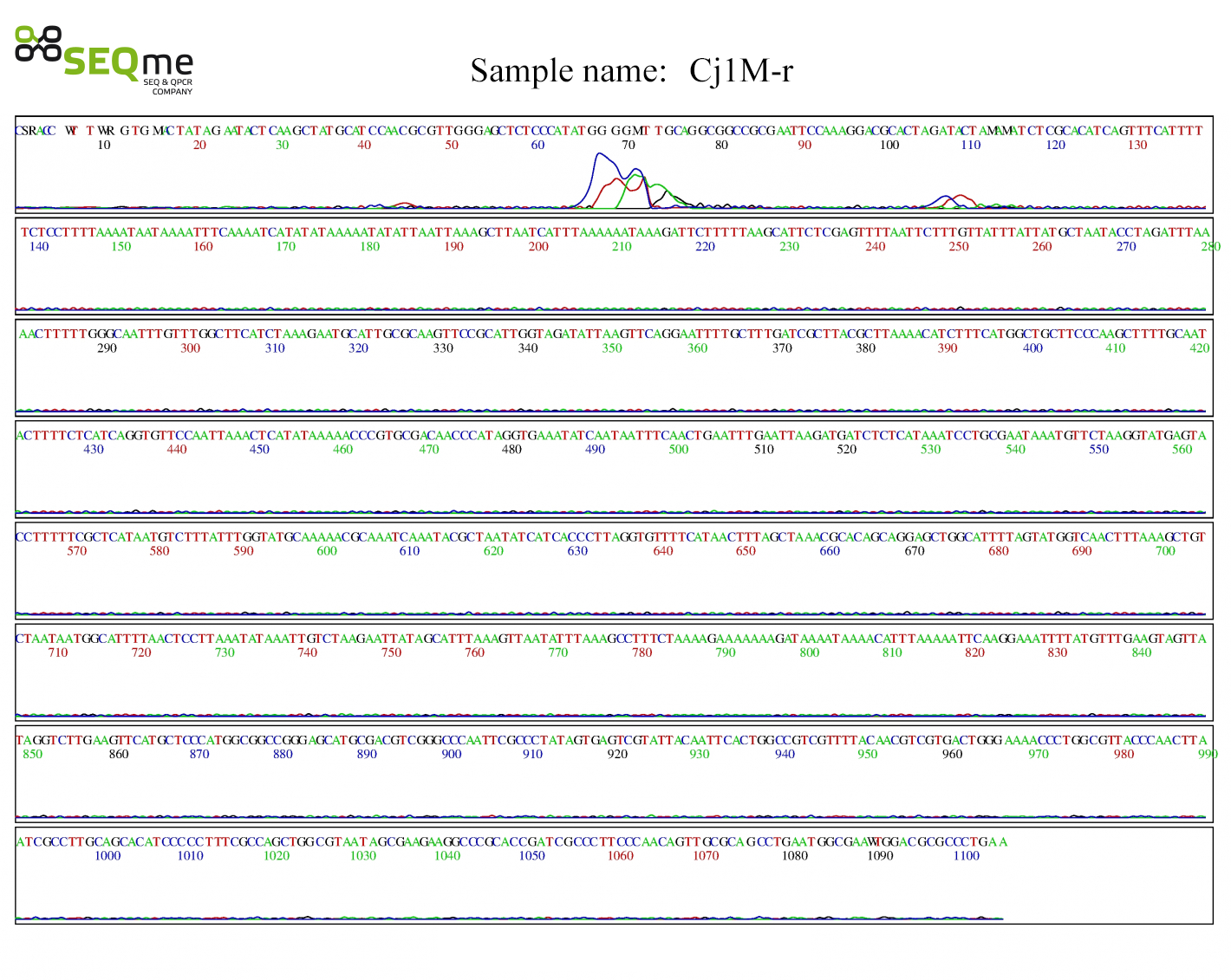

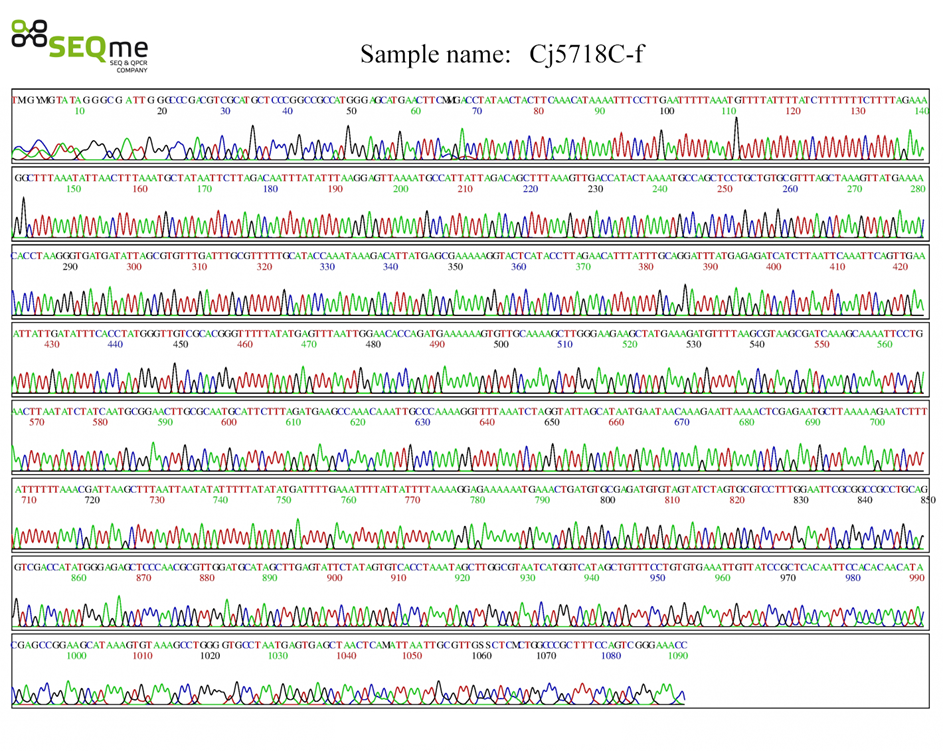


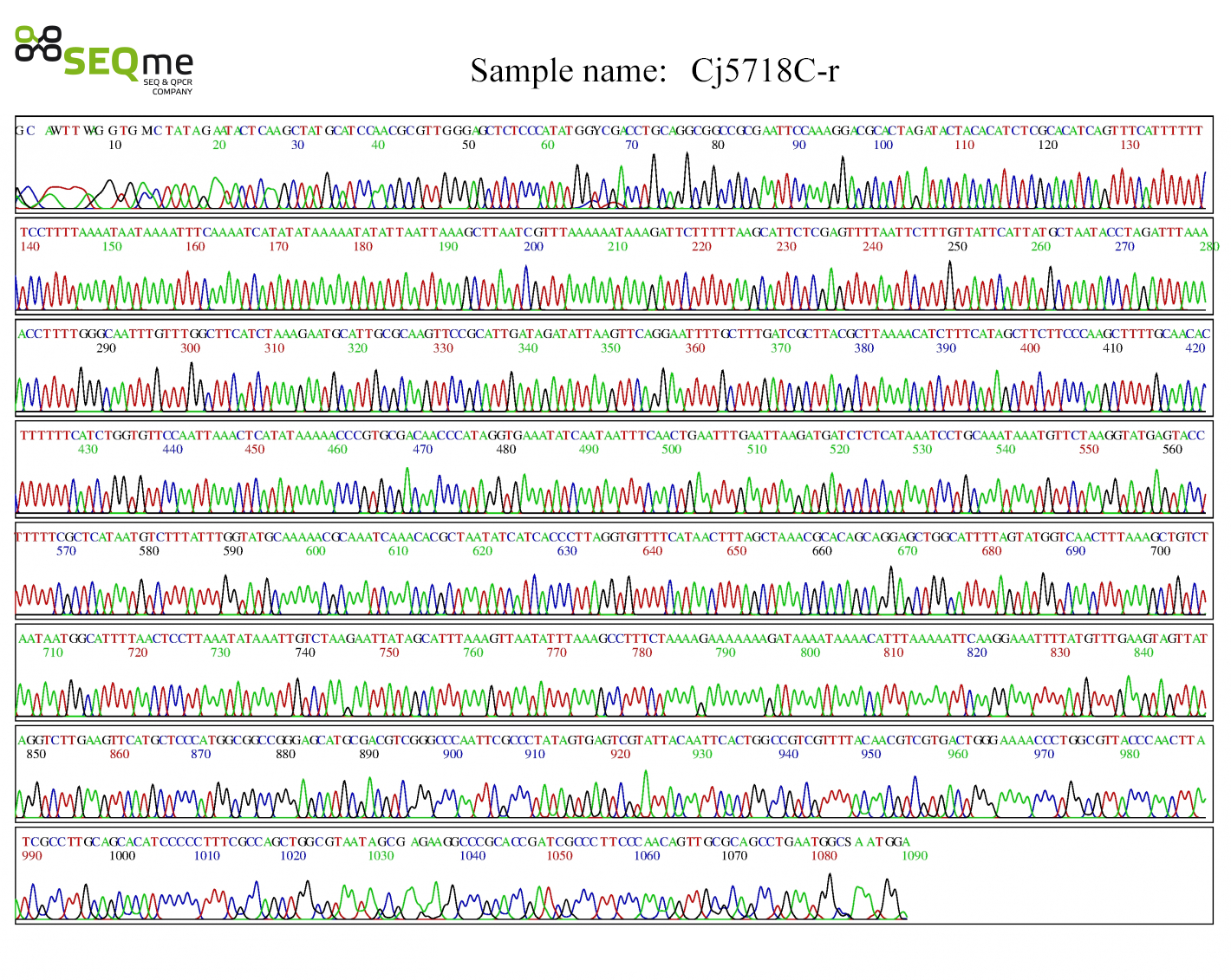


**Supplementary figure 2.** The nucleotide sequences obtained after Sanger sequencing of cloned fragments containing the *luxS* gene of each tested isolate of *C. jejuni.* 11168 (GenBank accession no. NC_002163.1) and *C. jejuni* 81-176 (GenBank accession no. GenBank accession no. CP000538.1).
